# Development and evaluation of analytical strategies for the monitoring of per- and polyfluoroalkyl substances from lithium-ion battery recycling materials

**DOI:** 10.1007/s00216-025-06165-8

**Published:** 2025-11-11

**Authors:** Emelie Meiers, Juliane Scholl, Morten Droas, Christian Vogel, Peter Leube, Thomas Sommerfeld, Abbas Bagheri, Christian Adam, Andreas Seubert, Matthias Koch

**Affiliations:** 1 Department 1 – Analytical Chemistry and Reference Materials, Bundesanstalt für Materialforschung und -prüfung (BAM), Berlin, Germany; 2 Department of Chemistry, Philipps-Universität Marburg, Marburg an der Lahn, Germany; 3 Department of Food Chemistry and Toxicology, Technische Universität Berlin, Berlin, Germany; 4 Department 4 – Materials and the Environment, Bundesanstalt für Materialforschung und -prüfung (BAM), Berlin, Germany; 5 Faculty of Energy and Economics, Technische Universität Clausthal, Clausthal, Germany

**Keywords:** PFAS target analysis, HILIC, Lithium-ion battery recycling, Black mass, Fluorine mass balance

## Abstract

Per- and polyfluoroalkyl substances (PFAS) are well-known as “forever chemicals” and persistent pollutants released by different anthropogenic sources. The potential release of PFAS from accumulating electronic waste and lithium-ion battery (LIB) recycling activities has gained increasing attention in the past years. This creates a need for analytical methods tailored for the determination of PFAS out of environmental matrices related to the named activities or directly out of the concerned materials. In this work, analytical strategies for the monitoring of PFAS in LIB recycling materials were explored for a group of legacy perfluoro sulfonic- and carbonic acids (PFSA and PFCA) and of fluorinated sulfonylimides suspected to be LIB electrolyte ingredients. These analytical strategies comprehend PFAS target approaches with a herein optimized liquid chromatography tandem mass spectrometry (LC–MS/MS) method equipped with a HILIC (hydrophilic interaction liquid chromatography) column in combination with the TOP (total oxidizable precursor) assay and an adapted sample preparation method for high-matrix LIB recycling materials. The validated target method was applied to a set of LIB recycling materials: end-of-life batteries, black masses from hydrometallurgical recycling, and gas absorption solutions from thermal treatment of black masses as part of the recycling procedure. Investigation results show that the LIB industry can be connected to the release of both “LIB”-PFAS, such as the target sulfonylimides, and “already-legacy” PFAS, like the PFSA and PFCA. Especially, the presence of trifluoroacetic acid (TFA) as an emerging pollutant in every investigated LIB material type underlines the threat of PFAS emissions from LIB waste and recycling activities.

**Graphical Abstract:**

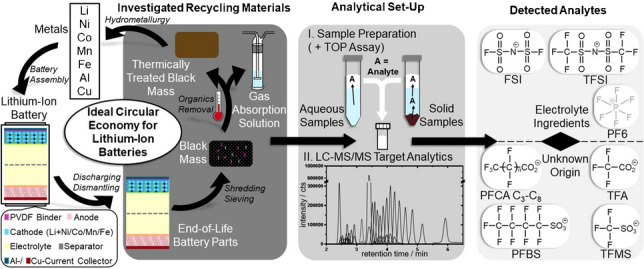

**Supplementary Information:**

The online version contains supplementary material available at 10.1007/s00216-025-06165-8.

## Introduction

Per- and polyfluoroalkyl substances (PFAS) present a group of persistent organic pollutants (POPs) that are defined by the presence of at least one perfluorinated methylene (-CF_2_) or methyl (-CF_3_) group according to the *Organisation for Economic Co-operation and Development* (OECD) [1]. By number, more than 4730 different PFAS with a variety of functional groups (carbonic acids, sulfonic acids, or alcohols) could be listed by the time of the OECD report in 2021 [1]. The release of PFAS from anthropogenic sources like Teflon® coatings, fire-fighting foams, or outdoor textiles is a matter of concern because of their environmental persistence and toxicity [2–4]. Although certain PFAS are subject to strict regulation by the Stockholm Convention and REACH (Registration, Evaluation, Authorisation, and Restriction of Chemicals) [5], there are certain applications that lean on their unique chemical properties: stability against harsh thermal and (electro)chemical conditions and repelling surface properties [6, 7]. The lithium-ion battery (LIB) industry represents one example of such a field of application [8]. With accumulating electronic waste and increasing battery recycling activities aiming to achieve a circular economy, the potential release of PFAS from such sources must be considered [8–10]. Especially, the hydrometallurgical recycling of LIBs may be a matter of concern. As this procedure consists of preparation steps like battery dismantling and shredding, yielding the so-called *black mass* powder that needs to undergo thermal treatment around 400–500 °C for organics removal to make metals from the battery accessible for hydrometallurgy (= leaching in aqueous solution to obtain metal salts), it applies conditions that may not be harsh enough to fully mineralize PFAS [11–14]. In this context, analytical methods adapted to concerned LIB or environmental matrices are needed. However, the analysis of PFAS originating from LIB matrices is challenged by uncertainties about exact ingredients. Approaches towards PFAS analysis from LIBs must therefore take the complexity of the cell chemistry into account.

**Fig. 1:**
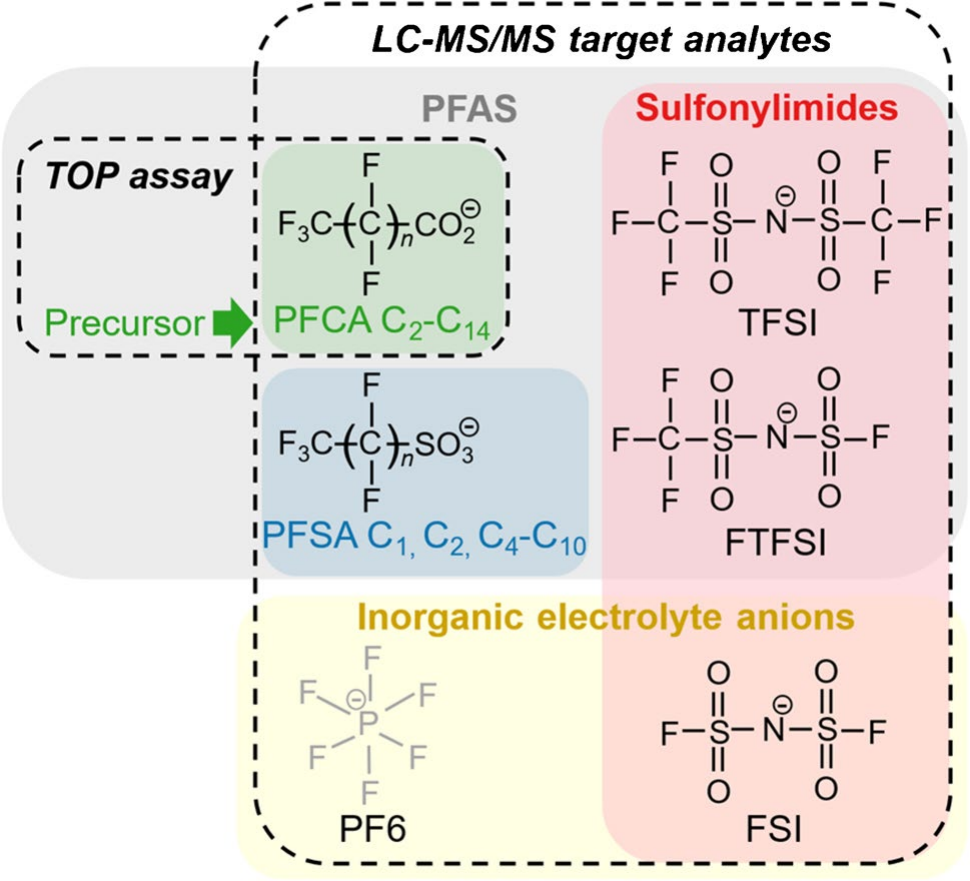
Overview of the analytical strategies for the portrayed analytes of this work. The LC–MS/MS target method includes the represented sulfonylimides, PFCA, and PFSA. The different chain lengths of the PFCA and PFSA are detailed in Table 1 with the corresponding number of C-atoms and *n*. The incorporation of the TOP assay into the analytical workflow allow to cover further PFAS which are precursor of the target PFCA. The hexafluorophosphate (PF6) anion is not related to PFAS and was not part of the original selection of target analytes of this work and is therefore represented in grey. The relevance of the PF6 anion will be discussed in following sections

The electrochemical reactions in a LIB rely on lithium ions that migrate between the anode and cathode and redox reactions that are mediated by transition metals on the cathode and often graphite-based materials on the anode [15, 16]. For proper function and performance, LIBs need an electrolyte allowing lithium-ion solvation and transport. The electrolyte itself is classically composed of a lithium salt with a weakly coordinating counter-anion, coordinating solvents like organic carbonates (i.e., ethylene carbonate or dimethyl carbonate) and additives [17, 18]. The most common counter-anion is the hexafluorophosphate (PF_6_^−^) anion, which is an inorganic molecular fluorinated species [19–21]. Further, PFAS anions like bis(trifluoromethanesulfonyl)imide (abbreviated as TFSI, bis-FMeSI or NTf2) [8, 22–24], (fluorosulfonyl)(trifluoromethanesulfonyl)imide (FTFSI) [23, 25], or the non-PFAS analogue bis(fluorosulfonyl)imide (FSI) [23, 25] are described (see Fig. 1 for structures). However, it is unknown if in commercial LIBs, the cited examples are used as part of the primary conducting salt with lithium as counter-cation, as ionic liquid solvents or as additives increasing battery performance, lifetime, and stability [8, 25–30]. Next to the named fluorinated anions, neutral fluorinated organic compounds (FOCs) like (poly)fluorinated carbonates, ethers, or alkylated phosphates can be incorporated in LIB electrolyte as solvents or additives [27, 31, 32]. Not all of them are defined as PFAS—like fluoroethylene carbonate (FEC) [32, 33]—but they also represent fluorine sources within the LIB and should therefore be considered in the PFAS discussion according to Rensmo et al*.* and Gao et al*.* [9, 34].

In LIBs, the anodic and the cathodic half-cell need to be separated by an isolating membrane. The most cited separator materials are polyethylene or polypropylene [35], but Song et al*.* also describe the possibility of using polymer electrolytes based on the fluorinated polymer poly(vinylidene fluoride) (PVDF) [36]. Usually, PVDF is used in LIBs as a binder material to increase cohesion on the cathode side [37–39]. Fluorinated polymers have the potential to liberate fluorinated degradation products [9, 40]. Cui et al*.* demonstrated that PTFE (polytetrafluoroethylene) and PVDF-based fluoropolymers can yield trifluoroacetic acid (TFA) during thermal decomposition at temperatures between 400 and 650 °C [40]. Consequently, the thermal treatment for organics removal preparing the hydrometallurgical recycling of LIBs can be suspected as a source of PFAS-related degradation products [9, 40, 41]. Next to the named sources potentially present in commercial LIBs, degradation processes during battery aging or induced by recycling steps could lead to a pool of fluorinated compounds—including PFAS—that can be released from LIB waste or recycling activities.

There are different analytical methods cited that focus on LIB-related emissions. However, the focus has formerly been on degradation products of the classical hexafluorophosphate electrolyte anion [19, 20, 42]. It is only since the last few years that the focus has shifted to PFAS, particularly from environmental matrices related to LIB waste and recycling activities [43]. Zhang et al*.* performed a study on the presence of PFAS, especially bis(perfluoroalkylsulfonyl)imides (bis-FASIs), in dust collected at or in the surroundings of electronic-waste (e-waste) sites in China [22]. They detected TFSI (= bis-FMeSI) in significant amounts – up to 12 ng/g in dust directly from an e-waste site [22]. Guelfo et al*.* studied the presence and impact of bis-FASIs and especially TFSI in environmental samples near production sites of bis-FASIs in the USA and Europe [8]. By investigating a LIB membrane, Guelfo et al*.* demonstrate the use of TFSI in commercial LIBs [8]. Continuously, different legacy perfluorosulfonic and carbonic acids (PFSA and PFCA) and other PFAS were found by Qi et al*.* within the non-target screening of environmental samples collected near a LIB-recycling park in China [10]. Similar findings are reported by Baqar et al*.* for the screening of PFAS from soil samples near an e-waste recycling site in Pakistan [44] and by Chen et al*.* for the non-target analysis of water or sediment samples related to LIB waste or recycling sites in China [43]. Baqar et al*.* additionally outline the dominant prevalence of TFSI and precursors of TFA [44]. Chen et al*.* conclude that the origin of PFAS suspected to be LIB-related requires confirmation by directly investigating LIB cells and materials [43].

Regarding methodology, all cited examples conducted their studies based on liquid chromatography mass spectrometry (LC–MS or LC–MS/MS) instrumentation, with most of them using C_18_-columns [8, 10, 22, 43–45]. Baqar et al*.* additionally applied a column with ion-exchanging properties to include TFA and other short-chain PFAS in the method [44]. One commonly used sample preparation method applies a methanolic extraction and a solid-phase extraction (SPE) clean-up with washing steps to elute undesired polar components, which discriminate against short-chain PFAS [46–50]. Thus, PFAS target analysis requires further developments to include polar short-chain (C_1_-C_3_) representatives in standardized sample preparation and LC–MS/MS instrumentation [51–53]. This is of special interest in the context of PFAS originating from LIBs, as short-chain PFAS like TFA can be expected from degradation processes in the cell or during recycling [40, 44]. In complement to target analysis, PFAS can be monitored via fluorine sum parameter measurements – accessible through combustion ion chromatography (CIC) [48, 54, 55]. Sum parameter measurements are, however, linked to uncertainties about the exact chemical nature of the fluorinated species [55]. Another tool in PFAS analysis is the total oxidizable precursor (TOP) assay, which transforms fluorinated precursor substances into the respective PFCA through oxidative conversion using hydroxyl radicals, thus allowing a broader monitoring  [51, 56, 57].

In this study, we propose a target method for the determination of PFSA (number of carbons: C_1_, C_2_, C_4_–C_10_), PFCA (number of carbons: C_2_–C_14_), the bis-FASI TFSI, and the structurally related sulfonylimides FTFSI and FSI directly out of LIB recycling materials. The analytical target method consists of a tailored sample preparation method, instrumentation via LC–MS/MS instrumentation equipped with an HILIC (hydrophilic interaction liquid chromatography) column, and quantifications via an isotope standard (ISTD) dilution procedure. To complement the target method, TOP assay and CIC fluorine sum parameter measurements were performed. Resulting evaluations could contribute to potential future regulations concerning PFAS in LIBs.

## Materials and methods

### Standards and chemicals

#### Extraction agents, TOP assay reagents, and LC–MS/MS solvents

For all experiments (except for the CIC measurements), only ultrapure water (< 0.055 µS/cm at 25 °C) produced with a Purelab® Flex 2 from Veolia Water Technologies AG (Celle, Germany) was used. Acetonitrile (ACN, for LC–MS), methanol (MeOH, for LC–MS), sodium carbonate (Na_2_CO_3_, anhydrous, for analysis), concentrated hydrochloric acid (HCl, 35–38%), sodium chloride (NaCl, for analysis), potassium peroxydisulfate (K_2_S_2_O_8_; for analysis), and sodium hydroxide (NaOH; for analysis) were supplied by Chemsolute®, Th. Geyer GmbH & Co. KG (Renningen, Germany). Formic acid (FA, for LC–MS) and ammonium formate (NH_4_FA, for LC–MS) were obtained from Carlo Erba Reagents GmbH (Emmendingen, Germany). Methyl *tert*-butyl ether (MtBE, for gas chromatography ECD and FID) was purchased from SupraSolv®, Merck KGaA (Darmstadt, Germany), and concentrated sulfuric acid (H_2_SO_4_, 98%, for analysis) from Emsure®, Merck KGaA (Darmstadt, Germany).

#### LC–MS/MS standards

Trifluoroacetic acid (TFA/PFCA C_2_, 101 μg/mL in methanol) and pentafluoroethanesulfonic acid (PFEtS/PFSA C_2_, 101 μg/mL in acetonitrile) were purchased from AccuStandard Inc. (New Haven, CT, USA). Pentafluoropropionic acid (PFPrA/PFCA C_3_, ≥ 97%), bis(trifluoromethane)sulfonimide lithium salt (LiTFSI, ≥ 99.95%), potassium hexafluorophosphate (KPF_6_, ≥ 99%), and lithium hexafluorophosphate electrolyte (LiPF_6_, 1 M in EC/DMC) were obtained from Merck KGaA/Sigma-Aldrich (St. Louis, MN, USA). Lithium (fluorosulfonyl)(trifluoromethanesulfonyl)imide (LiFTFSI, ≥ 97%) and sodium trifluoromethanesulfonate (NaTFMS/PFSA C_1_, ≥ 98%) were supplied by BLDpharm Ltd. (Cincinatti, OH, USA). Lithium bis(fluorosulfonyl)imide (LiFSI, ≥ 99.5%) was purchased from Carl Roth GmbH & Co. KG (Karlsruhe, Germany). A native PFCA and PFSA mix PFAC30PAR (PFCA C_4_-C_14_, PFSA C_4_-C_10_, see Table SI.1 in the SI for analyte levels, in methanol/6% (v/v) isopropanol) and an isotopically labeled PFCA and PFSA mix MPFAC-24ES (PFCA C_4_–C_12_ and C_14_, PFSA C_4_, C_6_, and C_8_; see Table SI.1 in the SI for analyte levels, in methanol/2% (v/v) isopropanol) were obtained from Wellington Laboratories Inc. (Guelph, Canada). Isotopically labeled sodium trifluoroacetate (99% ^13^C_2_-TFA, 50 μg/mL in methanol) was supplied by Cambridge Isotope Laboratories Inc. (Tewksbury, MA, USA).

Solid reference substances were weighed with a Cubis® II laboratory balance from Sartorius AG (Göttingen, Germany) and were dissolved in MeOH to produce stock solutions for further dilution with MeOH or MtBE. Each solvent addition for stock solution production and for the dilution of produced stock solutions or purchased standards was controlled gravimetrically. All stock solutions and diluted standards were stored at −18 °C and used for 5 months maximally.

#### CIC standards and chemicals

Ammonium fluoride hydrate (NH_4_F × 0.5 H_2_O, ≥ 99.995%) for the calibration of the CIC system was purchased from Puratronic™, Thermo Fisher Scientific Inc./AlfaAeser (Haverhill, MA, USA). The internal standard methanesulfonic acid (MeSO_3_H, ≥ 99.5%) and an IC multi-element standard ROTI®Star 7 anions (F^−^ 5 mg/L, Cl^−^ 10 mg/L, Br^−^ 25 mg/L, NO_2_^−^ 15 mg/L, NO_3_^−^ 25 mg/L, PO_4_^3−^ 40 mg/L, SO_4_^2−^ 30 mg/L in water) for counter-checking of fluoride concentrations at the IC system were obtained from Carl Roth GmbH & Co. KG (Karlsruhe, Germany). Argon and oxygen gas (both 5.0) for the combustion unit were supplied by Linde plc (Dublin, Ireland). The gas absorption unit and the IC system ran on ultrapure water (< 0.055 µS/cm at 25 °C) produced with a Milli-Q® EQ 7000 Ultrapure Water Purification System from Merck KGaA (Darmstadt, Germany). Ammonia (NH_3_, 25%) for the preparation of the alkaline methanesulfonic acid internal standard dilution was obtained from Suprapur®, Merck KGaA (Darmstadt, Germany). All dilutions for the aqueous solutions were performed volumetrically.

### Samples

#### End-of-life lithium-ion batteries

Commercial end-of-life pre-disposals were investigated, among that one round cell (LIB1) and two coin cells (LIB2, LIB3) providing voltages of 3 V. The cell chemistry and state of the cells are unknown. All batteries were discharged with an IT8211 Digital Control electronic load (ITECH Electronics Co., Ltd., New Tapei, China) to ≤ 2.0 V using the constant current mode. The discharge current was set to half of the electrical charging capacity in ampere hours. Subsequently, the cells were dismantled, opened, and specified battery parts (i.e., cathode, anode, separator, or combinations) were sampled for investigation.

#### Black masses

Lithium-ion battery black masses (BM) prepared for hydrometallurgical recycling were supplied by an industrial cooperation partner. The battery type represented in the black mass was exclusively lithium iron phosphate (LFP). Two distinct batches of equally prepared black masses (BM1 and BM2) were used for the investigations. The preparation of the black masses consisted of the discharging and dismantling of the end-of-life LFP batteries, vacuum distillation, mechanical shredding, and fractionation by particle size (< 500 µm). No thermal treatment for organics removal had yet been applied to the samples BM1 and BM2. A thermally treated black mass (BM1.T) was supplied by the same cooperation partner and prepared from the batch of the BM1 sample via thermal treatment at 600 °C under air to mass constancy. All samples were stored under ambient conditions before sample preparation.

#### Gas absorption solutions from thermal treatment of black mass

The black mass for thermal treatment was obtained from the industrial partner as described in the previous section. The industrial thermal treatment process for organics removal was simulated with a pendulum tube furnace (Carbolite Gero, Neuhausen, Germany) equipped with a pendulum device (Panasonic Corporation, Kadoma, Japan) and a heating system (Eurotherm Germany GmbH, Limburg, Germany). For each batch, 100 g of LFP black mass (BM1) was disposed of in the quartz glass reactor, and the pendulum intensity was set to the device-specific step “5.” The gas inlet of the reactor was connected to a compressed air valve set to a gas flow of 3 L/min. At the outlet of the reactor, a connection to a gas absorption bottle containing 150 mL of water for each batch was installed. For thermal treatment, the heating rate was set to 20 °C/min, and the final temperature (500 °C for batch GA1 and 250 °C for batch GA2) was held for 1 h. The gas absorption solutions were stored at 4 °C until sample preparation.

### Sample preparation

The sample preparation method is based on a procedure for the extraction of TFA from soil samples published by Scholl et al*.* [58]. Herein, the analyte is extracted via leaching out of the high matrix soil with an alkaline aqueous solution followed by salting out and acidifying to achieve the phase transfer of the analyte from the aqueous phase into an organic solvent (i.e., ethyl acetate or MtBE) in a one-pot procedure. This procedure was adapted to the matrices investigated in this work and differentiated into a qualitative approach for the end-of-life LIB parts or a quantitative approach for black masses and gas absorption solutions.

#### Qualitative procedure for end-of-life lithium-ion batteries

The sampled LIB parts were prepared in 15 mL polypropylene (PP) falcon tubes in a non-quantitative manner (no sample weighing was performed). As the addition of water and acidifying in the presence of the LIB parts led to harsh reactions between the acid and active battery material, the one-pot extraction from Scholl et al*.* [58] was adapted. First, 3 mL MtBE was added to the sample, and the mixture was horizontally shaken (10 min; 300 min^−1^; HS 501 D digital horizontal shaker, IKA-Werke GmbH & Co. KG, Staufen, Germany). The MtBE extract was transferred into a fresh 15-mL PP falcon tube and washed with 6 mL water, which was saturated with NaCl (approx. 5 g) and acidified with concentrated HCl (30 µL). The samples were then centrifuged (10 min; 4000 rpm; rcf 3828 g; 22 °C; 6K15 centrifuge, Sigma Laborzentrifugen GmbH, Osterode, Germany), and the MtBE layer was transferred into a PP PFAS-free vial, sealed with PFAS-free polyimide sealings, and injected for LC–MS/MS measurement or stored at −18 °C until measurement.

#### Quantitative procedure for black masses and gas absorption solutions

The samples were disposed into a 15-mL PP falcon tube, respectively, 0.5 g for black mass samples and 10 mL for aqueous gas absorption solutions under gravimetric control of the exact amount in both cases. For quantitative analysis, the samples were spiked with 0.1 mL of an ISTD mix (Table SI.2 in the SI) containing isotopically labeled TFA, the PFCA C_4_–C_12_ and C_14_, and the PFSA C_4_, C_6_, and C_8_ under gravimetric control to access the exact ISTD amount per sample weight. In the case of a matrix spike with native analyte for recovery calculations, 0.6 mL of a native analyte mix (Table SI.2 in the SI) was added to the samples in the same manner as described for the ISTD spike.

Black mass samples were extracted with 6 mL of an aqueous Na_2_CO_3_ solution (0.2% (w/w)) by ultrasonication (10 min; Sonorex AK100, Bandelin electronic GmbH & Co. KG, Berlin, Germany) and horizontal shaking (10 min; 300 min^−1^). The pre-extracted black mass samples and the untreated water (10 mL) samples were overlayered with MtBE (black mass: 4 mL, water: 2 mL), saturated with NaCl (black mass: approx. 1.5–2 g, water: approx. 2.5–3 g), and acidified with concentrated H_2_SO_4_ (black mass: 200 µL, water: 230 µL). When the quantification via the ISTD dilution was not possible, the exact amount of added MtBE was also controlled gravimetrically. Subsequently, the samples were horizontally shaken (10 min; 300 min^−1^) and centrifuged (10 min; 4000 rpm; rcf 3828 g; 22 °C).

For the water samples, the MtBE phase was directly transferred into a PP PFAS-free vial, sealed with PFAS-free polyimide sealings, and injected for measurement or stored at −18 °C until. For the high matrix black mass samples, an aliquot of 2 mL of the MtBE layer was transferred into a fresh 15-mL falcon tube and washed with 2 mL of diluted H_2_SO_4_ (18% (w/w)) by manual shaking for 10 s. The aqueous phase was saturated with NaCl (1 g), and the samples were centrifuged (10 min; 4000 rpm; rcf 3828 g; 22 °C). Then, the MtBE phase was transferred into a PP PFAS-free vial, sealed with PFAS-free polyimide sealings, and injected for measurement or stored at −18 °C until measurement.

All MtBE extracts or dilutions could directly be injected into the LC–MS/MS system. For subsequent CIC measurements, aliquots of 1.2 mL of the MtBE extracts were dried under a gentle stream of nitrogen at room temperature, re-dissolved in 1.0 mL MeOH, and undilutedly injected into the CIC system. All dilution steps were controlled gravimetrically. The extracts for the CIC measurements were previously not spiked with ISTD or native standards to prevent falsification of the fluorine sum parameter by the spikes.

#### TOP (total oxidizable precursor) assay

For the solid black mass samples, the dTOP assay (direct TOP assay) was performed. For this, 0.1 g of the black mass sample was weighed into a 15-mL PP falcon tube. After the addition of 5.4 mL of water and 0.6 mL of a 30% NaOH solution, the samples were treated in an ultrasonic bath (5 min) and manually shaken. For the gas absorption solutions, 9 mL of the sample was dispensed into the 15-mL falcon tube, and 1 mL of 30% NaOH solution was added. In both cases, 0.1 g of K_2_S_2_O_8_ was added to the prepared samples, and the pH was controlled to assure a pH > 12. The samples were subsequently incubated in a drying cabinet (Memmert GmbH & Co. KG, Schwabach, Germany) at 85 °C for 6 h with a slightly open screw cap (to allow gas circulation). Every hour, the samples were shaken manually. After the reaction incubation time, the samples were cooled to 4 °C and again checked for a pH > 12. Before extraction, the samples were neutralized with concentrated H_2_SO_4_ and spiked with 0.1 mL of the ISTD mix (Table SI.2 in the SI). After this, the extraction was performed as described above for the black mass and water samples, respectively.

### LC–MS/MS target measurements

#### Instrumentation

The instrumental measurement method is developed from chromatographic conditions advertised by the producer of the HILIC column (Restek Corporation, Bad Homburg, Germany) and published by Liang et al*.* [52].

LC–MS/MS measurements were performed using an Agilent 1290 Infinity II LC system (Agilent Technologies Deutschland GmbH, Waldbronn, Germany) coupled to an Agilent 6495 C triple quadrupole MS (Agilent Technologies Deutschland GmbH, Waldbronn, Germany) equipped with an electrospray ionization (ESI) spray chamber (Agilent Jet Stream). The system was controlled by the Mass Hunter Workstation (version 10.1, Agilent Technologies Deutschland GmbH).

The injection volume was set to 2 µL and carried out with an automated sampler. Chromatographic separation was achieved with a Raptor Polar X HILIC column (2.1 mm × 50 mm; 2.7 µm particles; Restek Corporation, Bad Homburg, Germany) equipped with a Raptor Polar X guard column (2.1 mm × 5 mm; Restek Corporation, Bad Homburg, Germany). The column temperature was set to 40 °C and the flow rate to 0.5 mL/min. A mixture of an aqueous phase (A) composed of water with 0.05% (v/v) FA and 10 mM NH_4_FA and an organic phase (B) composed of ACN/MeOH in a volumetric ratio of 60:40 with 0.05% (v/v) FA was used as the mobile phase. For elution, a gradient program was applied: starting at a mobile phase composition of 95% B and 5% A, a linear gradient from 0 to 6 min increasing A from 5 to 15% was set. At 6.1 min, a step back to 5% A was inserted with a hold time until the end of the run at 8 min. Nitrogen was used as dry gas at a flow rate of 11 L/min and a temperature of 250 °C. The nebulizer pressure was set to 25 psi and the capillary voltage was set to 3 kV in negative ion mode. The detection was performed in a targeted dynamic multiple reaction monitoring (dMRM) mode with a 1 min retention time window for each analyte and a cycle time of 500 ms. The fragmentor voltage was fixed at 166 V. Table SI.3 in the SI contains the qualifier- and quantifier mass transitions for each native and isotopically labeled analyte, the collision energies, and retention times.

#### Data processing

The data processing and peak integration was carried out with Mass Hunter Quantitative Analysis (version 12.1, Agilent Technologies Deutschland GmbH). Quantifications were preferably performed based on an ISTD dilution calibration and an external calibration if the ISTD dilution variant failed (see Table SI.4 and SI.5 in the SI for calibration curve data). For analytes lacking a corresponding ISTD, a similar ISTD in chain length and retention time was used (see Table SI.2 for assignments of native analytes and ISTD). All calibration standards were diluted from a native standard mix (Table SI.2) with MtBE to a total volume of 4 mL and spiked with 0.1 mL of an ISTD mix (Table SI.2). Subsequently, the calibration standards were prepared in analogy to the black mass samples. Details on quantifications can be found in the equations SI.1 and SI.2 in the SI.

#### Quality assurance

To control for contaminations, solvent blanks and method blanks were spiked with the ISTD mix (Table SI.2) and prepared with each batch of samples. Instrumental blanks and carry-over effects were monitored by injecting eluent blanks. All samples were investigated in independent triplicates.

The determination method was validated, and all validation criteria were determined based on the described procedure for the high matrix black mass samples. Method trueness was approached as the mean matrix recovery of native analyte spikes on an uncontaminated black mass sample (BM1.T = “blank matrix”) for six independent replicates. The mean recovery variance of the blank matrix spikes was taken as the method precision parameter. Measurement precision was assessed by the percental standard deviation of the measured signals from tenfold injection of the highest and lowest calibration standard for each analyte and found to be under 14% at the lowest calibration standard level and under 5% at the highest calibration standard level. Limit of detection (LOD) and limit of quantification (LOQ) were calculated based on the blank method from DIN32645 [59], after assuring variance homogeneity, normal distribution, absence of outliers, and trends of the used data with an error probability of 5%. Table SI.6 in the SI contains the parameter for the statistical tests.

#### Adapted method incorporating the hexafluorophosphate anion

As the hexafluorophosphate anion (PF6) was originally not part of the selected PFAS-related targets but gained attention due to the results revealed in this work, it was treated apart from the other analytes. Additionally, within the above-described measurement method, co-elution and matrix effects occurred, leading to the need to develop a chromatographic approach for the determination of the hexafluorophosphate anion via an adapted version of the above-described HILIC-ESI–MS/MS method. The detailed instrumentation is given in the SI in section SI.3.1–2.

### CIC sum parameter measurements

#### Instrumentation

The CIC measurements were performed based on the works of Roesch et al*.* [48] and Vogel et al*.* [60]. The instrumentation was achieved by the coupling of an AQF-2100H combustion system (Mitsubishi Chemical Europe GmbH, Düsseldorf, Germany) controlled by the software NSX 2100 (version 10.2.5, Mitsubishi Chemical) to an ICS Integrion ion chromatographic (IC) system (Thermo Fisher Scientific GmbH, Dreieich, Germany) controlled by the software Chromeleon (version 7.2.10, Thermo Fisher Scientific GmbH). The combustion temperature was set to 1050 °C. The detailed description of the CIC instrumentation is given in section SI.1.2 in the SI.

#### Data processing

Data processing and peak area integration was performed using the software Chromeleon (version 7.2.10, Thermo Fisher Scientific GmbH). Quantifications were performed based on a nine-point external calibration curve (*R*^2^ = 0.99980) for fluoride at the levels: 0.00, 0.02, 0.04, 0.08, 0.14, 0.20, 0.40, 0.50, 1.00 mg/L. As calibration standards, aqueous solutions of NH_4_F were prepared, combusted, and measured with the same method as the samples. The dilution factor introduced by the gas absorption unit and monitored by the internal standard was respected for all quantifications. For further details on the calculations, see equation SI.3 in the SI.

#### Quality assurance

To check for contaminations, solvent blanks and method blanks were prepared with each batch of samples. Instrumental blanks were monitored by combusting and measuring the fluoride response of the unloaded ceramic boat in all four measurements and at least ten times per batch. All peak areas were corrected against the mean instrumental blank from the combustion of the unloaded boats. Each sample was measured in triplicates.

The concentrations of the manually prepared NH_4_F standards were checked on the IC side with a certified 7-anion standard containing fluoride. For this, the IC parameter corresponded to those described above.

## Results and discussion

Figure 1 and Table 1 contain overviews and details on the analytical strategies and target analytes of this work based on an LC–MS/MS instrumentation and the TOP assay to cover precursor yielding the respective PFCA after oxidative conversion.

**Table 1:** Explanation of the PFCA and PFSA target analytes by their abbreviations, number of perfluorinated methylene groups (*n*) and number of carbons

***n***	PFCA			PFSA		
0	C_2_	TFA	Trifluoroacetic acid	C_1_	TFMS	Trifluoromethanesulfonic acid
1	C_3_	PFPrA	Perfluoropropionic acid	C_2_	PFEtS	Perfluoroethanesulfonic acid
2	C_4_	PFBA	Perfluorobutanoic acid	C_3_	PFPrS	Perfluoropropanesulfonic acid
3	C_5_	PFPeA	Perfluoropentanoic acid	C_4_	PFBS	Perfluorobutanesulfonic acid
4	C_6_	PFHxA	Perfluorohexanoic acid	C_5_	PFPeS	Perfluoropentanesulfonic acid
5	C_7_	PFHpA	Perfluoroheptanoic acid	C_6_	PFHxS	Perfluorohexanesulfonic acid
6	C_8_	PFOA	Perfluorooctanoic acid	C_7_	PFHpS	Perfluoroheptanesulfonic acid
7	C_9_	PFNA	Perfluorononaoic acid	C_8_	PFOS	Perfluorooctanesulfonic acid
8	C_10_	PFDA	Perfluorodecanoic acid	C_9_	PFNS	Perfluorononanesulfonic acid
9	C_11_	PFUnDA	Perfluoroundecanoic acid	C_10_	PFDS	Perfluorodecanesulfonic acid
10	C_12_	PFDoDA	Perfluorododecanoic acid			
11	C_13_	PFTrDA	Perfluorotridecanoic acid			
12	C_14_	PFTDA	Perfluorotetradecanoic acid			

### LC–MS/MS target method

The developed LC–MS/MS target method includes PFSA (number of carbons: C_1_, C_2_, C_4_–C_10_), PFCA (number of carbons: C_2_–C_14_), TFSI, FTFSI, and the inorganic FSI. The given sulfonylimides qualify as target analytes due to reports of their use in LIBs [22–25]. The PFSA and PFCA were incorporated into the analysis because they are targeted by different existing regulations concerning PFAS [5]. Although there are no reports of the direct use of the given PFSA and PFCA in LIBs, especially short-chain representatives can be suspected to be related to degradation products of fluoropolymers during LIB cycling or recycling. Another aspect qualifying the PFCA as target analytes is their role as reaction products from PFAS precursor within the TOP assay [51, 56, 57].

**Fig. 2:**
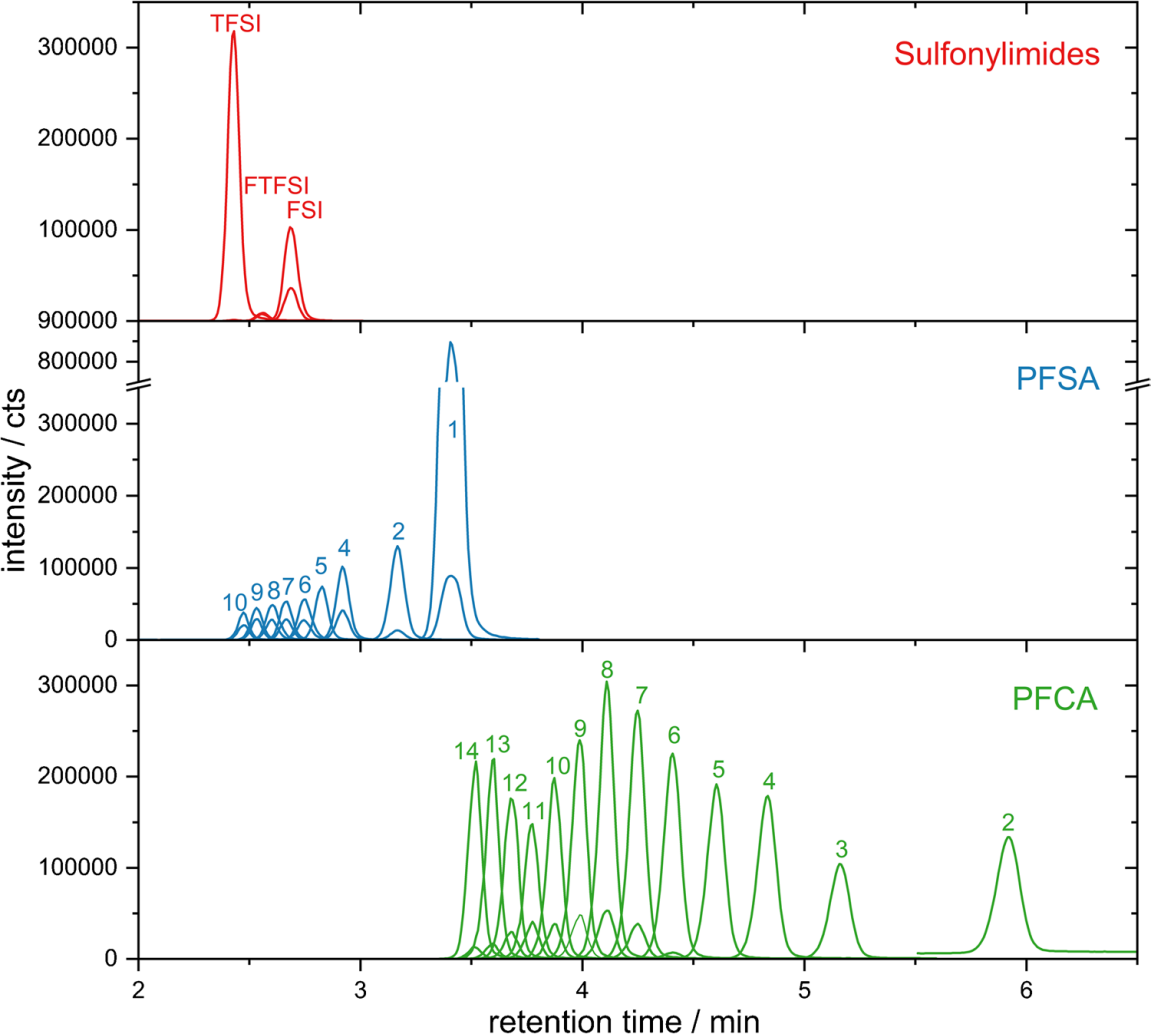
HILIC-ESI–MS/MS chromatogram of a standard mix containing the sulfonylimide, PFSA, and PFCA analytes. The numbers attributed to the PFSA and PFCA peaks indicate the number of carbon atoms of the chain. The standard mix consists of the undiluted stock solution mixture (Table SI.2) of the native analytes

Figure 2 shows the chromatogram obtained from the measurement of the target analytes in a mix standard with the HILIC-ESI–MS/MS setup. The analytes show HILIC separation behavior. Critical factors for the separation in the presented system are the ratio of aqueous and total organic parts (5:95 (v/v) initial ratio aqueous to organic for the gradient elution) and the ratio of methanol and acetonitrile (40:60 (v/v) ratio MeOH to ACN) in the organic part of the ternary mobile phase (see Fig. SI.1 with comments and section SI.3.1 in the SI for further insights). Variation in the retention times (± 0.4 min within one measurement day) represents the biggest and typical challenge within the application of HILIC methods. By setting the right retention time windows in the dynamic reaction monitoring detection mode, retention time drifts can be overcome. Although co-elution of analytes occurs, especially in the retention time regions between 2 and 3 min, selectivity and sensitivity of the method are assured due to the MS/MS detection including a quantifier and a qualifier mass transition for the concerned analytes.

### Method validation for quantitative analysis

The sample preparation procedure for the quantitative analysis of the black mass and gas absorption samples was optimized from the extraction method for TFA from soil samples published by Scholl et al*.* [58]. The optimization was conducted in terms of response recovery of the analytes after matrix spike (on BM1.T as “blank matrix”) and is detailed in section SI.2.2 in the SI.

To further assure the developed procedure regarding suitability for quantifications, for both an external and an ISTD dilution calibration, trueness and method precision were monitored via recovery and variance of native analyte spikes on blank matrix (BM1.T) to comment on method strengths or limitations with or without the use of ISTDs. In Table 2, the recoveries and variances for the external and the ISTD dilution calibration are displayed along with the calibration curve *R*^2^ values and the LOQs for the method for quantifications with the ISTD dilution calibration. Detailed calibration curve data for both procedures are shown in Tables SI.4 and SI.5 in the SI.

**Table 2:** Mean recovery and variance (*n* = 6) for six matrix spikes (on blank black mass) quantified with an external calibration vs. an ISTD dilution calibration with their *R*^2^ values and LOQs for the ISTD dilution variant

	External calibration			ISTD dilution calibration				
Analyte	*R*^2^	Mean recovery/%	Mean recovery variance/%	*R*^2^	Mean recovery/%		Mean recovery variance/%	LOQ*/ng/(mL MtBE-extract)
TFSI	0.99740	106	9	0.99055	107	4		0.001
FTFSI	0.99467	104	11	0.99436	104	8		0.019
FSI	0.99593	104	11	0.99423	106	7		0.005
PFDS	0.99868	102	9	0.99532	105	3		0.002
PFNS	0.99690	103	8	0.99617	105	4		0.005
PFOS	0.99613	103	11	0.99777	105	3		0.006
PFHpS	0.99586	108	12	0.98729	110	5		0.002
PFHxS	0.99816	106	10	0.99434	108	3		0.004
PFPeS	0.99301	104	9	0.99409	104	4		0.002
PFBS	0.99753	107	13	0.99932	105	6		0.001
PFEtS	0.99282	98	10	0.99459	98	5		0.006
TFMS	0.97151	69	9	0.99364	67	9		0.003
PFTDA	0.98780	100	9	0.99724	107	2		0.004
PFTrDA	0.99381	107	11	0.98919	100	4		0.020
PFDoDA	0.99084	114	14	0.99540	106	4		0.008
PFUnDA	0.98060	125	14	0.99700	102	4		0.019
PFDA	0.99033	116	15	0.99771	105	5		0.025
PFNA	0.98945	110	13	0.99600	109	2		0.019
PFOA	0.98365	106	12	0.99917	105	3		0.022
PFHpA	0.93931	78	8	0.98745	105	2		0.018
PFHxA	0.99435	28	3	0.99775	111	3		0.032
PFPeA	0.99039	22	4	0.99483	108	3		0.026
PFBA	0.99539	30	29	0.99587	104	3		0.031
PFPrA	0.99539	26	36	0.98980	79	26		0.023
TFA	0.99581	94	22	0.99707	114	3		0.116

*The LOD is, respectively, the given LOQ divided by three. The presented LOQs in ng/(mL extract) can be calculated for each particular case by multiplying with the MtBE extraction volume and by dividing by the sample amount used for extraction (black mass: 4 mL MtBE, 0.5 g sample; water: 2 mL MtBE, 10 mL sample)

The external and internal calibration procedures both result in acceptable recoveries between 67 and 116% for the listed analytes above, including PFHpA. For the PFCA beginning at PFHxA (C_6_) until TFA (C_2_) via the external calibration method, the recoveries are low (< 30%) and/or linked to high variances. Irreproducible matrix effects that are especially pronounced for the smaller PFCA with low masses increased peak broadening (compare Fig. 2), and only one detectable mass transition may represent the cause of this. The quantification via the corresponding ISTD compensates for matrix effects, leading to recoveries around 100% for PFHxA, PFPeA, PFBA, and TFA. However, in the case of PFPrA, the corresponding ISTD was not available, and isotopically labeled PFBA was used instead. This eliminates the ability to monitor all variations and matrix effects impacting the detection of PFPrA, which explains the relatively low recovery even via the ISTD dilution calibration and the low method precision (variance of 26%). The same applies for TFMS with 67% recovery quantified via isotopically labeled PFBS. Thus, especially for the concerned short-chain PFAS, the use of the structurally corresponding ISTD plays a major role in compensating for matrix effects and achieving reliable quantification without systematic underreporting. Globally, the *R*^2^ values and method precision parameters are better with the ISTD dilution procedure. For these reasons, the ISTD dilution procedure is superior to the external calibration for the ability to monitor variations, i.e., due to matrix effects, extraction inefficiency, and instrumental drifts. Therefore, the ISTD dilution calibration was applied for the following quantifications, if possible. The respective LOQs of the analytes based on the ISTD dilution procedure reach low levels (parts-per-trillion order of magnitude), confirming the method sensitivity.

### Application to LIB recycling materials and implications

Table 3 displays the results from the investigations of the LIB recycling materials with the target method for the overall detected analytes. The investigated materials are discharged and dismantled end-of-life LIB parts (LIB1–3), LFP black masses prepared for the hydrometallurgical recycling (BM1–2), and gas absorption solutions (GA1–2) from the thermal treatment of one LFP black mass at two different temperatures to simulate potential PFAS emissions from the process used for organics removal prior to hydrometallurgy. As the materials concern different steps of the recycling and/or waste management chain for LIBs, the investigations enable insights into potential PFAS emissions within a bigger frame. At the same time, the value of the developed target analytical method can be tested regarding possible applications. For the end-of-life LIB parts, only qualitative findings are reported. For the black masses (BMs) and gas absorption solutions (GAs), quantification results are indicated.

**Table 3:** Qualitative (for end-of-life LIB parts) and quantitative (for black masses and gas absorption solutions) findings for the investigations of LIB recycling materials

Material	Detected analytes/ng/(g sample)^a^				
	TFSI	FSI^b^	PFBS	TFMS	TFA
LIB1_(cathode, separator)	Yes	Yes	-	-	Yes
LIB2_(combined cell parts)	Yes	Yes	-	Yes	Yes
LIB3_(cathode)	Yes	Yes (traces)	-	-	Yes + all PFCA C_3_-C_8_
LIB3_(separator, electrode parts^c^)	-	Yes (traces)	-	-	Yes + all PFCA C_3_-C_8_
BM1	-	320 ± 7	7 ± 1	-	-
BM1.T	-	-	-	-	-
BM2	-	750 ± 30	6 ± 1	-	4.3 ± 0.1
GA1	-	-	-	-	(0.6 ± 0.1) < LOQ
GA2	-	-	-	-	1.1 ± 0.2

^a^All quantified analyte levels are based on independently prepared triplicates and reported as the protonated analyte form unless indicated differently

^b^Quantified in the black mass samples via an external calibration due to high levels. The quantifications are reported as the deprotonated analyte form

^c^Optically, it was not distinguishable if the electrode residues on the separator were cathodic or anodic elements

#### Qualitative investigation of end-of-life lithium-ion batteries

In extracts from parts of all three investigated end-of-life LIBs, the sulfonylimides TFSI and FSI were found. The presence of TFSI and FSI indicates that they may be used as electrolyte additives or primary conducting salts in commercial LIBs, as previously described in the literature [8, 23, 25]. Although the investigations of the LIB extracts were performed in a non-quantitative manner, peak intensities indicate that TFSI represents the dominant analyte when present. This is in line with the reporting of Guelfo et al*.*, where TFSI was also found dominant in different commercial LIBs [8]. Further to the use of TFSI in the electrolyte, Guelfo et al*.* also discuss the possibility of TFSI as a PVDF binder additive [8, 61] which would entrain a local distribution of TFSI in the cell and could explain the absence of TFSI in the “separator, electrode parts” extract of LIB3 while being present in the cathode part extract.

All investigated extracts contained TFA, which is suspected to be a degradation product from aging processes of fluorinated LIB components (PVDF binder or FOC additives), rather than originating from the direct use in the cell and therefore present independent of the specific cell type, chemistry, or investigated part. Further, the LIB2 cell extract contained TFMS, and both extracts from LIB3 tested positive for all PFCA C_3_-C_8_. It is not clear whether these PFAS compounds are related to aging processes and degradation products or to a direct use in the LIB, i.e., as electrolyte or fluoropolymer binder additives. As they are present in distinct cells only, it is possible that they are part of a particular cell chemistry.

Especially the presence of the named PFCA is intriguing because some of them (PFHxA (C_6_), PFHpA (C_7_), and PFOA (C_8_)) are already under regulation [5]. This underlines the need for monitoring LIB-related PFAS emissions in the future—focusing on LIB-PFAS and already-legacy PFAS.

#### Quantitative investigation of black masses

Both investigated black mass samples from LFP type batteries before thermal treatment contained high levels of FSI (> 300 ng/(g sample)) and trace levels of PFBS (6–7 ng/(g sample)). The black mass BM2 additionally showed TFA contamination at trace levels (4 ng/(g sample)). The presence of FSI and TFA in the black masses can be explained under the same aspects as discussed for the end-of-life LIB parts. The provenance of PFBS in the LFP cells is not clear. For both materials, the TOP assay did not lead to the detection of more analytes or higher levels, respectively.

The thermal treatment of the black mass BM1 within the hydrometallurgical recycling workflow removes FSI and PFBS, leading to the material BM1.T being uncontaminated with any of the target analytes. This removal of (organic) fluorinated compounds is desirable for the recycling process and hydrometallurgy efficiency [62] but is connected to the risk of the release of toxic compounds to the environment. The subsequent paragraph, therefore, discusses the findings for gas absorption solutions from the thermal treatment of the material BM1.

#### Quantitative investigation of gas absorption solutions from thermal treatment of black mass

The gas absorption solutions GA1 and GA2 distinguish themselves by the temperature used for the thermal treatment of the material BM1 (contaminated with PFBS and FSI), 500 °C, and 250 °C, respectively. The chosen temperatures frame a reasonable range compared to reports of the thermal treatment step prior to hydrometallurgy [12, 14]. Both GAs contained exclusively TFA at trace levels (0.5–1 ng/(g sample)). This is in line with the expectation that TFA may be released as a thermal degradation product from fluoropolymers like the LIB binder polymer PVDF [40] or as a degradation product from other fluorinated compounds present in the black mass. The application of the TOP assay to the GAs yielded an increase in the determined TFA level by the factor of 60 for GA1 and by the factor of 15 for GA2. This indicates that TFA and unknown TFA precursors are released in significant orders of magnitude during the thermal treatment of the black mass (sum of TFA and precursor 7.5–60 g per ton black mass). With an estimated 8 million tons of LIB waste generated from electric vehicles alone by 2040 [63], even the hydrometallurgical recycling of a small percentage of this waste with pretreatments as investigated in this study is linked to a significant release of TFA (precursor). Especially against the background of the German Environment Agency’s recommendation for the TFA level in drinking water to be under 60 µg/L [64], the important release of TFA from LIB recycling activities represents a threat.

For the thermal treatment at 250 °C, the obtained thermally treated black mass material and residing powder in the reactor were still tested positively for FSI and PFBS, which underlines the influence of the temperature on the removal efficiency. At 500 °C thermal treatment, the materials in the reactor showed no positive hit for any target analyte. However, the presence of TFA and TFA precursor in the respective GA shows that a temperature of 500 °C is not sufficient for full mineralization of PFAS-related compounds in the black mass. The thermal treatment of LIB black masses prior to hydrometallurgy may therefore be a source of PFAS emissions to the environment.

### Black mass samples: critical assessment of the results and further approaches

#### Fluorine sum parameter and mass balance

As the black masses were quantitatively extractable and present the interface between LIB ingredients and PFAS release within the recycling process, further investigations were performed on the materials. The objective was to get more insights on the importance of the selected PFAS targets and to critically assess the implications of the findings. For this purpose, a fluorine sum parameter measurement was performed via combustion ion chromatography, and the determined PFAS values were balanced with the fluorine mass sum. Table 4 contains the fluorine sum parameter for the extracts of the black mass materials BM1, BM1.T, and BM2 and presents the fluorine mass balance weighing the sum parameter and the levels of the quantified fluorinated target analytes (FSI, PFBS, and TFA).

**Table 4:** Mass balance of the target analytes and the extractable fluorine sum parameter for the black mass materials

Material	Extractable fluorine sum parameter^a^/ng/(g sample)	Fluorine equivalents^b^/ng/(g sample)			Non-explainable extractable fluorine/%
		FSI^c^	PFBS	TFA	
BM1	4600 ± 50	68 ± 1	4.0 ± 0.6	-	98.4
BM1.T	177 ± 6	-	-	-	n.a.^d^
BM2	8500 ± 80	157 ± 6	3.2 ± 0.6	0.308 ± 0.007	98.1

^a^The fluorine sum parameter is calculated based on one independent sample extraction with three-fold injection. The values are corrected against the instrument blank but not against the method blank which is at 297 ± 18 ng/(g sample) extractable fluorine

^b^All quantified analyte levels are based on independently prepared triplicates and reported as the protonated analyte form unless indicated differently. The fluorine equivalents respect the loss of analyte due to the drying and re-uptake in MeOH of the extracts for the CIC sum parameter measurements

^c^Quantified in the black mass samples via an external calibration due to high levels. The quantifications are reported as the deprotonated analyte form

^d^Not applicable, because the fluorine sum parameter is below the method blank indicating that the sample can be considered as uncontaminated with extractable fluorine

The black masses before thermal treatment show important extractable fluorine levels; meanwhile, the thermally treated material BM1.T shows less fluorine than the method blank and can therefore be considered as uncontaminated. If applicable, the target analytes cover less than 2% of the respective extractable fluorine sum. The mass balance gap indicates that unknown fluorinated compounds and potentially other PFAS-related substances are behind the main part of the extractable fluorine sum parameter. To approach this question, the respective extracts were checked with low resolution (LR) MS-scans using the HILIC-ESI–MS/MS method. Detected masses and mass transitions revealed that the hexafluorophosphate (PF6) anion and related hydrolysis products known from degradation pathways discussed in the literature may be present in the black mass extracts [42, 65]. Hexafluorophosphate is the anion in the classical LiPF_6_ conducting salt used in LIBs and is in relation to a broad spectrum of toxic hydrolysis products [19, 65]. This is why the following paragraph deviates from the original aim of this study, focusing on PFAS, and gives a short introduction to prospectives for the incorporation of PF6 and related species into target analysis for LIB materials.

### Opening the focus: LC–MS/MS approaches incorporating the PF_6_^−^ anion and hydrolysis products

LC–MS/MS approaches incorporating first the PF6 anion and secondly the hydrolysis products were explored to provide evidence for their importance in the context of LIB recycling. As only a reference standard for the PF6 anion itself was available in this work, detected hydrolysis products are postulated based on the comparison of their low-resolution mass transitions to literature data of known PF6 degradation products and pathways [65]. As these findings require further confirmation, all details are represented in section SI.3.4 in the SI. Thus, the key findings are briefly resumed in the following paragraph, representing a potential point of departure for future studies.

The challenge encountered with the determination of the PF6 anion with the original HILIC-ESI–MS/MS method used for the PFAS and sulfonylimides of this work was the co-elution with FSI and matrix effects in extracts of black mass samples. This necessitated the adaptation of the chromatographic parameter to achieve separation of FSI and PF6 on the Raptor Polar X HILIC column. Separation was achieved as represented in Fig. SI.3 and as detailed in the sections SI.3.1 and SI.3.2 in the SI.

Following the method optimization, an external calibration for PF6 could be established (see section SI.3.3 in the SI). However, the quantification of PF6 in the concerned extracts led to an increase in the explainable fluorine sum parameter by only 2.7% at best (see Table SI.11 in the SI). Although PF6 is present at high levels in the original sample extracts, the drying step necessary for the CIC sum parameter measurements caused significant losses of PF6 which must be accounted for in the mass balance. The high tendency to hydrolysis and degradation of PF6 makes the aqueous extraction followed by harsh acidifying combined with the drying step for CIC measurement used in this work unsuitable for unfalsified quantification of PF6 from matrix. Thus, different potentially PF6-related hydrolysis product signals could be detected in LR-MS scans—before and after drying of the extracts with modified relative peak intensities—that performed mass transitions to m/z 79 (PO_3_^−^) and m/z 63 (PO_2_^−^) indicating the presence of phosphate structures like PO_2_F_2_^−^ (difluorophosphate) or PO_2_OMeF^−^ (methyl fluorophosphate) (see Fig. SI.4 and Table SI.12 in the SI). This opens the focus to another group of fluorinated compounds—notably fluorinated phosphates or phosphoric acid esters—potentially released in LIB recycling activities next to PFAS. However, the stated findings must be controlled by using reference standards for the concerned phosphates or via high-resolution mass spectrometry measurements. If the quantification of the named species is aimed at, alternative and less harsh extraction methods must be explored.

## Conclusion

In this work, a target analytical method for some legacy PFAS (PFSA and PFCA) and LIB-related (PFAS)-sulfonylimides based on HILIC-ESI–MS/MS instrumentation was developed for application to materials related to LIB recycling. The HILIC separation allows for the examination of long-chain and short-chain representatives of the PFSA and PFCA in one analytical setup, while selectivity can easily be adapted through mobile phase conditions. This closes a gap in the analysis of PFAS and allows quick screenings of samples covering an interesting range of analytes.

Sample preparation was achieved through a simple and quick extraction method centered around a liquid–liquid extraction step to obtain the target analytes in an organic layer (i.e., MtBE), allowing separation from saline matrix components, adaptable for water and solid samples. The advantages of the use of an ISTD dilution calibration were demonstrated for quantification with the developed method, leading to high accuracy and low LOQs (parts-per-trillion order of magnitude).

The application of the method to the LIB materials—end-of-life LIB parts or black masses—led to the detection of important intensities of TFSI and FSI pointing to their use in commercial batteries. Further, traces of PFAS like PFBS, TFMS, and the PFCA (C_3_–C_8_) could be detected depending on the material. The reason for the presence of those PFAS could not be elucidated in this work. Further, TFA was present in all investigated materials themselves. Additionally, associated unknown precursors were present in the gas absorption solutions. This points out the potential of PFAS and especially TFA emission from LIB waste and recycling activities. To address the question of whether some of the detected analytes represent LIB additives or aging-related degradation products, systematic studies of LIB cell chemistries before and after aging are necessary.

Within a bigger frame of fluorinated compounds, the target analytes cover only a minor percentage of the detected fluorine sum parameter extracted from the black mass samples. Further elucidation of the fluorine sum parameter pointed out the importance of another group of analytes in the context of LIBs: the hexafluorophosphate anion— known from the classical application as LiPF_6_ conducting salt in the LIB electrolyte—and related hydrolysis products like fluorinated phosphates or fluorinated phosphoric acid esters. As the LIB industry is under constant development, nowadays’ end-of-life batteries can differ from future end-of-life batteries regarding the electrolyte chemistry, electrolyte additives, and binder additives. Therefore, systematic investigations of LIB ingredients and cell chemistries, including all intriguing substance classes, i.e., PFAS or (fluorinated) phosphoric acid esters, threatening to be emitted during waste management and recycling are needed. The application of non-target approaches could be a possibility to perform non-biased investigations to further explore LIB chemistries.

## Supplementary Information

Below is the link to the electronic supplementary material.

Supplementary Material 1 (DOCX 1.78 MB)

## Data Availability

All data and material is available.
