# Supplementary material for: Development and evaluation of analytical strategies for the monitoring of per- and polyfluoroalkyl substances from lithium-ion battery recycling materials
Source: Anal Bioanal Chem. 2025 Nov 11;417(29):6567–83. doi: 10.1007/s00216-025-06165-8 (PMC12641044; doi:10.1007/s00216-025-06165-8)
Supplement: Supplementary file 1 — Supplementary Material 1 (DOCX 1.78 MB) [file 216_2025_6165_MOESM1_ESM.docx]

**Supporting Information**

**Development and Evaluation of Analytical Strategies for the Monitoring of Per- and Polyfluoroalkyl Substances from Lithium-Ion Battery Recycling Materials**

Emelie Meiers^1,2^, Juliane Scholl^1,3^, Morten Droas^4,5^, Christian Vogel^4^, Peter Leube^4^, Thomas Sommerfeld^1^, Abbas Bagheri^1^, Christian Adam^4^, Andreas Seubert^2^, Matthias Koch^1*^

*^1^ Bundesanstalt für Materialforschung und -prüfung (BAM); Department 1 – Analytical Chemistry and Reference Materials; Berlin, Germany*

*^2^ Philipps-Universität Marburg; Department of Chemistry; Marburg an der Lahn, Germany*

*^3^ Technische Universität Berlin; Department of Food Chemistry and Toxicology; Berlin, Germany*

*^4^ Bundesanstalt für Materialforschung und -prüfung (BAM); Department 4 – Materials and the Environment; Berlin, Germany*

*^5^ Technische Universität Clausthal; Faculty of Energy and Economics; Clausthal, Germany*

* Correspondence: matthias.koch@bam.de

**Table of Contents**

[**SI.1 Experimental Back-Up Information** 1](#_Toc202782130)

[**SI.1.1 LC-MS/MS Target Measurements** 1](#_Toc202782131)

[**SI.1.2 CIC Sum Parameter Measurements** 6](#_Toc202782132)

[**SI.2 Target Method Development for PFAS and Sulfonylimides** 7](#_Toc202782133)

[**SI.2.1 HILIC-ESI-MS/MS Chromatographic Conditions** 7](#_Toc202782134)

[**SI.2.2 Extraction Procedure** 8](#_Toc202782135)

[**SI.3 LC-MS/MS Instrumentation Incorporating the Hexafluorophosphate Anion** 10](#_Toc202782136)

[**SI.3.1 Method Development for HILIC-ESI-MS/MS with the Raptor Polar X Column** 10](#_Toc202782137)

[**SI.3.2 Instrumentation of the Final Method via HILIC-ESI-MS/MS with the Raptor Polar X Column** 11](#_Toc202782138)

[**SI.3.3 Quantifications and Updated Fluorine Mass Balance Including Hexafluorophosphate** 11](#_Toc202782139)

[**SI.3.4 Hexafluorophosphate Related Fluorinated Degradation Products** 12](#_Toc202782140)

## **SI.1 Experimental Back-Up Information**

### **SI.1.1 LC-MS/MS Target Measurements**

Table SI.1 contains information on the used native PFCA and PFSA mix (PFAC30PAR) and the isotopically labeled mix (MPFAC-24ES) purchased from Wellington Laboratories (Guelph, Canada). It is to note that the purchased mixtures contain more PFAS analytes than those indicated in the table below. However, only information on the analytes relevant in this work is displayed. All analytes are further referred to by their abbreviations without specification of the stereodescriptor (L) or without indication of the sum symbol in case of mixtures of branched and linear isomers because reported findings are not influenced by this.

**Table SI.1** Analyte levels in the purchased PFAS mix standards PFCA30PAR and MPFAC-24ES from Wellington Laboratories

| **PFAC30PAR (native mix)** | | | **MPFAC-24ES (ISTD mix)** | | |
| --- | --- | --- | --- | --- | --- |
| Analyte | Level salt (K/Na) / ng/mL | Level protonated acid / ng/mL | ISTD | Level salt (Na) / ng/mL | Level protonated acid / ng/mL |
| L-PFDS | 1000 (Na) | 965 | ^13^C_8_-PFOS |  |  |
| L-PFNS | 1000 (Na) | 962 |  |  |  |
| ∑ PFOS* | 999 (K) | 928 |  | 1000 | 958 |
| L-PFHpS | 1000 (Na) | 953 | ^13^C_3_-PFHxS | 1000 | 948 |
| ∑ PFHxS* | 1000 (K) | 914 |  |  |  |
| L-PFPeS | 1000 (Na) | 941 | ^13^C_3_-PFBS |  |  |
| L-PFBS | 1000 (K) | 887 |  | 1000 | 932 |
| PFTDA | / | 1000 | ^13^C_2_-PFTDA | / | 1000 |
| PFTrDA | / | 1000 | ^13^C_2_-PFDoDA |  |  |
| PFDoDA | / | 1000 |  | / | 1000 |
| PFUnDA | / | 1000 | ^13^C_7_-PFUnDA | / | 1000 |
| PFDA | / | 1000 | ^13^C_6_-PFDA | / | 1000 |
| PFNA | / | 1000 | ^13^C_9_-PFNA | / | 1000 |
| PFOA | / | 1000 | ^13^C_8_-PFOA | / | 1000 |
| PFHpA | / | 1000 | ^13^C_4_-PFHpA | / | 1000 |
| PFHxA | / | 1000 | ^13^C_5_-PFHxA | / | 1000 |
| PFPeA | / | 1000 | ^13^C_5_-PFPeA | / | 1000 |
| PFBA | / | 1000 | ^13^C_4_-PFBA | / | 1000 |

* Sum of branched and linear isomers.

Table SI.2 displays the analyte levels of the native and ISTD mix standards used for all tests and quantifications with the HILIC-ESI-MS/MS method for PFSA, PFCA und sulfonylimides. The native analytes are listed according to the ISTD used for quantifications.

**Table SI.2** Analyte levels in the prepared native and ISTD mix standards used for the LC-MS/MS experiments

| **Native mix in MtBE** | | | **ISTD mix in MtBE** | |
| --- | --- | --- | --- | --- |
| Analyte | Level / ng/(g solvent) | ISTD used for quantification | ISTD | Level / ng/(g solvent) |
| PFDS | 17.6 | ^13^C_8_-PFOS | ^13^C_8_-PFOS |  |
| PFNS | 17.5 | ^13^C_8_-PFOS |  | 62.0 |
| PFOS | 16.9 | ^13^C_8_-PFOS |  |  |
| PFHpS | 17.4 | ^13^C_3_-PFHxS | ^13^C_3_-PFHxS | 61.3 |
| PFHxS | 16.7 | ^13^C_3_-PFHxS |  |  |
| PFPeS | 17.2 | ^13^C_3_-PFBS | ^13^C_3_-PFBS | 60.2 |
| PFBS | 16.2 | ^13^C_3_-PFBS |  |  |
| PFEtS | 15.1 | ^13^C_3_-PFBS |  |  |
| TFMS * | 144.9 | ^13^C_3_-PFBS |  |  |
| TFSI * | 15.7 | ^13^C_3_-PFBS |  |  |
| FTFSI * | 53.5 | ^13^C_3_-PFBS |  |  |
| FSI * | 54.9 | ^13^C_3_-PFBS |  |  |
| PFTDA | 18.2 | ^13^C_2_-PFTDA | ^13^C_2_-PFTDA | 64.9 |
| PFTrDA | 18.2 | ^13^C_2_-PFDoDA | ^13^C_2_-PFDoDA | 64.9 |
| PFDoDA | 18.2 | ^13^C_2_-PFDoDA |  |  |
| PFUnDA | 18.2 | ^13^C_7_-PFUnDA | ^13^C_7_-PFUnDA | 64.9 |
| PFDA | 18.2 | ^13^C_6_-PFDA | ^13^C_6_-PFDA | 64.9 |
| PFNA | 18.2 | ^13^C_9_-PFNA | ^13^C_9_-PFNA | 64.9 |
| PFOA | 18.2 | ^13^C_8_-PFOA | ^13^C_8_-PFOA | 64.9 |
| PFHpA | 18.2 | ^13^C_4_-PFHpA | ^13^C_4_-PFHpA | 64.9 |
| PFHxA | 18.2 | ^13^C_5_-PFHxA | ^13^C_5_-PFHxA | 64.9 |
| PFPeA | 18.2 | ^13^C_5_-PFPeA | ^13^C_5_-PFPeA | 64.9 |
| PFBA | 18.2 | ^13^C_4_-PFBA | ^13^C_4_-PFBA | 64.9 |
| PFPrA | 16.4 | ^13^C_4_-PFBA |  |  |
| TFA | 52.3 | ^13^C_2_-TFA | ^13^C_2_-TFA | 70.1 |

* Analyte levels applicable for the deprotonated anion. In all other cases, the protonated acid form is concerned.

In table SI.3, the retention times and the MS/MS parameter for the detection of the native analytes and ISTDs with the HILIC-ESI-MS/MS method for PFCA, PFSA and sulfonylimides are given.

**Table SI.3** Detection parameter for the PFCA, PFSA and sulfonylimide analytes

|  |  | **Quantifier transition** | | **Qualifier transition** | |
| --- | --- | --- | --- | --- | --- |
| Native analytes | Retention time ± 0.3 / min | m/z → m/z | Collision energy / eV | m/z → m/z | Collision energy / eV |
| TFSI | 2.4 | 280 → 80 | 43 | 280 → 64 | 43 |
| FTFSI | 2.6 | 230 → 83 | 43 | 230 → 64 | 43 |
| FSI | 2.7 | 180 → 83 | 43 | 180 → 64 | 43 |
| PFDS | 2.4 | 599 → 80 | 63 | 599 → 99 | 55 |
| PFNS | 2.6 | 549 → 80 | 59 | 549 → 99 | 51 |
| PFOS | 2.6 | 499 → 80 | 52 | 499 → 99 | 48 |
| PFHpS | 2.7 | 449 → 80 | 48 | 449 → 99 | 44 |
| PFHxS | 2.7 | 399 → 80 | 48 | 399 → 99 | 43 |
| PFPeS | 2.8 | 349 → 80 | 40 | 349 → 99 | 36 |
| PFBS | 2.9 | 299 → 80 | 43 | 299 → 99 | 36 |
| PFEtS | 3.2 | 199 → 80 | 43 | 199 → 99 | 43 |
| TFMS | 3.4 | 149 → 80 | 43 | 149 → 99 | 43 |
| PFTDA | 3.5 | 713 → 669 | 12 | 713 → 219 | 28 |
| PFTrDA | 3.6 | 663 → 619 | 11 | 663 → 269 | 24 |
| PFDoDA | 3.7 | 613 → 569 | 11 | 613 → 319 | 20 |
| PFUnDA | 3.8 | 563 → 519 | 11 | 563 → 269 | 19 |
| PFDA | 3.9 | 513 → 469 | 8 | 513 → 219 | 16 |
| PFNA | 4.0 | 463 → 419 | 8 | 463 → 219 | 16 |
| PFOA | 4.1 | 413 → 369 | 7 | 413 → 169 | 19 |
| PFHpA | 4.2 | 363 → 319 | 7 | 363 → 169 | 19 |
| PFHxA | 4.4 | 313 → 269 | 7 | 313 → 119 | 23 |
| PFPeA | 4.6 | 263 → 219 | 7 | / | / |
| PFBA | 4.8 | 213 → 169 | 7 | / | / |
| PFPrA | 5.2 | 163 → 119 | 7 | / | / |
| TFA | 5.9 | 113 → 69 | 7 | / | / |
| ISTDs | Retention time ± 0.3 / min | m/z → m/z | Collision energy / eV | m/z → m/z | Collision energy / eV |
| ^13^C_8_-PFOS | 2.6 | 507 → 80 | 52 | 507 → 99 | 48 |
| ^13^C_3_-PFHxS | 2.7 | 402 → 80 | 48 | 402 → 99 | 43 |
| ^13^C_3_-PFBS | 2.9 | 302 → 80 | 43 | 302 → 99 | 36 |
| ^13^C_2_-PFTDA | 3.5 | 715 → 670 | 12 | 715 → 169 | 36 |
| ^13^C_2_-PFDoDA | 3.7 | 615 → 570 | 11 | 615 → 269 | 20 |
| ^13^C_7_-PFUnDA | 3.8 | 570 → 525 | 11 | 570 → 270 | 19 |
| ^13^C_6_-PFDA | 3.9 | 519 → 474 | 8 | 519 → 219 | 16 |
| ^13^C_9_-PFNA | 4.0 | 472 → 427 | 8 | 472 → 223 | 16 |
| ^13^C_8_-PFOA | 4.1 | 421 → 376 | 7 | 421 → 172 | 19 |
| ^13^C_4_-PFHpA | 4.2 | 367 → 322 | 7 | 367 → 169 | 19 |
| ^13^C_5_-PFHxA | 4.4 | 318 → 273 | 7 | / | / |
| ^13^C_5_-PFPeA | 4.6 | 268 → 223 | 7 | / | / |
| ^13^C_4_-PFBA | 4.8 | 217 → 172 | 7 | / | / |
| ^13^C_2_-TFA | 5.9 | 115 → 70 | 7 | / | / |

Table SI.4 shows the ISTD dilution calibration curve data for the PFCA, PFSA and sulfonylimide analytes. Table SI.5 shows the external calibration curve data for the PFCA, PFSA and sulfonylimide analytes.

**Table SI.4** Calibration curve data for the ISTD dilution procedure for all analytes with the HILIC-ESI-MS/MS procedure and after preparation of the calibration standards according to the sample preparation

| Native analytes | Number of calibration points | Calibration curve slope  *a*_ISTD_ | Calibration curve *y*-intersection *b*_ISTD_ | Calibration curve *R*^2^ | Level lowest calibration point / ng/(g solvent) in MtBE | Level highest calibration point / ng/(g solvent) in MtBE |
| --- | --- | --- | --- | --- | --- | --- |
| TFSI * | 8 | 2.610 | 0.254 | 0.99055 | 0.1 | 7.5 |
| FTFSI * | 8 | 0.027 | 0.005 | 0.99436 | 0.4 | 25.4 |
| FSI * | 8 | 0.092 | 0.027 | 0.99423 | 0.4 | 26.2 |
| PFDS | 8 | 0.634 | 0.068 | 0.99532 | 0.1 | 8.4 |
| PFNS | 8 | 0.776 | 0.061 | 0.99617 | 0.1 | 8.4 |
| PFOS | 8 | 0.923 | 0.058 | 0.99777 | 0.1 | 8.1 |
| PFHpS | 8 | 0.681 | 0.079 | 0.98729 | 0.1 | 8.3 |
| PFHxS | 8 | 0.832 | 0.086 | 0.99434 | 0.1 | 7.9 |
| PFPeS | 8 | 0.690 | 0.059 | 0.99409 | 0.1 | 8.2 |
| PFBS | 8 | 0.933 | 0.057 | 0.99932 | 0.1 | 7.7 |
| PFEtS | 8 | 1.029 | 0.059 | 0.99459 | 0.1 | 7.2 |
| TFMS * | 8 | 0.285 | -0.335 | 0.99364 | 1.0 | 69.0 |
| PFTDA | 8 | 0.777 | 0.062 | 0.99724 | 0.1 | 8.7 |
| PFTrDA | 8 | 1.170 | 0.126 | 0.98919 | 0.1 | 8.7 |
| PFDoDA | 8 | 0.908 | 0.071 | 0.99540 | 0.1 | 8.7 |
| PFUnDA | 8 | 0.937 | 0.006 | 0.99700 | 0.1 | 8.7 |
| PFDA | 8 | 0.974 | 0.057 | 0.99771 | 0.1 | 8.7 |
| PFNA | 8 | 0.891 | 0.118 | 0.99600 | 0.1 | 8.7 |
| PFOA | 8 | 0.988 | 0.061 | 0.99917 | 0.1 | 8.7 |
| PFHpA | 8 | 0.922 | 0.106 | 0.98745 | 0.1 | 8.7 |
| PFHxA | 8 | 0.885 | 0.113 | 0.99775 | 0.1 | 8.7 |
| PFPeA | 8 | 0.916 | 0.093 | 0.99483 | 0.1 | 8.7 |
| PFBA | 8 | 0.828 | 0.071 | 0.99587 | 0.1 | 8.7 |
| PFPrA | 8 | 0.564 | 0.081 | 0.98980 | 0.1 | 7.8 |
| TFA | 8 | 0.734 | 0.069 | 0.99707 | 0.4 | 24.9 |

* Analyte levels applicable for the deprotonated anion. In all other cases, the protonated acid form is concerned.

**Table SI.5** External calibration data for all analytes with the HILIC-ESI-MS/MS procedure and after preparation of the calibration standards according to the sample preparation

| Native analytes | Number of calibration standards | Calibration curve slope *a*_external_ / (cts·s·g)/ng | Calibration curve *y*-intersection *b*_external_ / cts·s | Calibration curve *R*^2^ | Level lowest calibration point / ng/(g solvent) in MtBE | Level highest calibration point / ng/(g solvent) in MtBE |
| --- | --- | --- | --- | --- | --- | --- |
| TFSI * | 8 | 44296 | -3617 | 0.99740 | 0.1 | 7.5 |
| FTFSI * | 8 | 454 | -239 | 0.99467 | 0.4 | 25.4 |
| FSI * | 8 | 1564 | -518 | 0.99593 | 0.4 | 26.2 |
| PFDS | 8 | 4943 | -41 | 0.99868 | 0.1 | 8.4 |
| PFNS | 8 | 6041 | -360 | 0.99690 | 0.1 | 8.4 |
| PFOS | 8 | 7199 | -531 | 0.99613 | 0.1 | 8.1 |
| PFHpS | 8 | 7154 | -361 | 0.99586 | 0.1 | 8.3 |
| PFHxS | 8 | 8753 | -485 | 0.99816 | 0.1 | 7.9 |
| PFPeS | 8 | 11721 | -1603 | 0.99301 | 0.1 | 8.2 |
| PFBS | 8 | 15063 | -931 | 0.99753 | 0.1 | 7.7 |
| PFEtS | 8 | 17463 | -2439 | 0.99282 | 0.1 | 7.2 |
| TFMS * | 8 | 4847 | -16571 | 0.97151 | 1.0 | 69.0 |
| PFTDA | 8 | 43003 | -7687 | 0.98780 | 0.1 | 8.7 |
| PFTrDA | 8 | 40835 | -4597 | 0.99381 | 0.1 | 8.7 |
| PFDoDA | 8 | 31774 | -5675 | 0.99084 | 0.1 | 8.7 |
| PFUnDA | 8 | 17960 | -4190 | 0.98060 | 0.1 | 8.7 |
| PFDA | 8 | 15866 | -2414 | 0.99033 | 0.1 | 8.7 |
| PFNA | 8 | 16773 | -725 | 0.98945 | 0.1 | 8.7 |
| PFOA | 8 | 20095 | 2342 | 0.98365 | 0.1 | 8.7 |
| PFHpA | 6 | 18032 | 15510 | 0.93931 | 0.1 | 8.7 |
| PFHxA | 6 | 40684 | 3973 | 0.99435 | 0.1 | 4.9 |
| PFPeA | 8 | 31367 | 1363 | 0.99039 | 0.1 | 8.7 |
| PFBA | 8 | 27912 | -958 | 0.99539 | 0.1 | 8.7 |
| PFPrA | 8 | 19026 | 944 | 0.99539 | 0.1 | 7.8 |
| TFA | 8 | 8930 | -2043 | 0.99581 | 0.4 | 24.9 |

* Analyte levels applicable for the deprotonated anion. In all other cases, the protonated acid form is concerned.

The calculation of the analyte level per sample weight via the ISTD dilution calibration is represented in equation SI.1:

| $Level\left( native analyte \right)=\frac{Peak area ratio\left( \frac{native analyte}{spiked ISTD} \right)-b_{\mathrm{ISTD}}}{a_{\mathrm{ISTD}}}\cdot\frac{Quantity\left( spiked ISTD \right)}{Sample weight}$ | **Eq.** SI.1 |
| --- | --- |

The calculations for the external calibration are shown in equation SI.2:

| $Level\left( native Analyte \right)=\frac{Peak area\left( native analyte \right)-b_{\mathrm{external}}}{a_{\mathrm{external}}}\cdot\frac{Extraction solvent weight}{Sample weight}$ | **Eq.** SI.2 |
| --- | --- |

Herein, the variables and their units are as follows:

| [*Level*(native analyte)] | ng/(g sample) |
| --- | --- |
| [*Peak area ratio*] | / |
| [*a*_ISTD_] | / |
| [*b*_ISTD_] | / |
| [*Quantity*(spiked ISTD)] | ng |
| [*Sample weight*] | g |
| [*Peak area*] | cts·s |
| [*a*_external_] | (cts·s·g)/ng |
| [*b*_external_] | cts·s |
| [*Extraction solvent weight*] | g |

Table SI.6 contains the statistical testing parameter for the target method validation. Here included are tests for outliers, variance homogeneity, normal distribution, and trends. The tests were applied to the dataset used for the LOD and LOQ determination according to the blank method from DIN32645 based on tenfold (*n*=10) measurements of the method blank, the lowest and the highest calibration standard with an error probability of 5 %. If the test statistic fulfills the given condition, the test leads to a positive result. For normal distribution and variance homogeneity, a positive result is wanted and for the trend and outlier test, a negative result is wanted.

**Table SI.6** Statistical test parameters for the target method validation and LOD/LOQ determination according to DIN32645

| Test | Test statistic *λ* | Condition for positive result |
| --- | --- | --- |
| Normal distribution | (*x*_max_ – *x*_min_) / *s* | 2.67 < *λ* < 4.24 |
| Trend (Neumann) | ∑(*x*_i_-*x*_i+1_)^2^ / (*f* · *s*^2^) | *λ* < 1.062 |
| Variance homogeneity | if *s*_lowestCal_ > *s*_highestCal_: *s*^2^_lowestCal_ / *s*^2^_highestCal_ | *λ* < 3.179 |
|  | if *s*_lowestCal_ < *s*_highestCal_: *s*^2^_highestCal_ / *s*^2^_lowestCal_ |  |
| Outlier (Grubbs) | \|*x*_mean_ - *x*\| / *s* | *λ* > 2.176 |

*x* – measured value; *s* – mean standard deviation; *f* – degrees of freedom, defined as *n*-1 with *n* – number of repetitions

### **SI.1.2 CIC Sum Parameter Measurements**

The CIC measurements were performed based on works of Roesch *et al.* [1] and Vogel *et al.* [2]. The instrumentation was achieved by the coupling of an AQF-2100H combustion system (Mitsubishi Chemical Europe GmbH, Düsseldorf, Germany) controlled by the software NSX 2100 (Version 10.2.5, Mitsubishi Chemical) to an ICS Integrion ion chromatographic (IC) system (Thermo Fisher Scientific GmbH, Dreieich, Germany) controlled by the software Chromeleon (version 7.2.10, Thermo Fisher Scientific GmbH).

For injection, 250 µL of the sample dissolved in MeOH were drawn from the vial and loaded onto a ceramic boat by an autosampler (ASC-210). The ceramic boat was packed with glass wool (low in fluorine, long-fibered A1) and pretreated for 15 min at the combustion temperature before use to remove organic contaminations. Within one batch, the unloaded ceramic boat was treated with the combustion program each four measurements to reduce carry-over effects. The combustion unit (HF-210) was kept at 1050 °C and operated with an argon carrier gas flow rate of 150 mL/min, an oxygen combustion gas flow rate of 300 mL/min and an argon water supply flow rate of 100 mL/min (system specific intensity “2”). The detailed program of the combustion is given in table SI.7. In the gas absorption unit (GA-210) for the combustion gases, 5 mL of an aqueous internal standard solution (MeSO_3_H 2.2 mg/L and 0.15 mM NH_3_) were pre-dispensed and filled up with water to a total volume of approximatively 18 mL at the end of a combustion. The exact absorption volume for each sample was determined based on the dilution of the MeSO_3_H from the pre-dispensed internal standard and respected for quantifying calculations.

The IC system was equipped with a potassium hydroxide eluent-generator (Dionex EGC 500 KOH), a continuously regenerated trap column (Dionex CR-TC 600), an autosampler (Dionex AS-AP), a Dionex IonPac AS20 analytical column (2 mm x 250 mm; 7.5 µM particles) with a Dionex IonPac AG20 guard column (2 mm x 50 mm) and a Dionex DRS600 dynamically regenerated suppressor (all Thermo Fisher Scientific GmbH, Dreieich, Germany). The sample loop for injection of the solution from the gas absorption unit had a volume of 100 µL. The column temperature was set to 30 °C and the flow rate at 0.25 mL/min. Elution was performed with a gradient given in table SI.8. The conductivity detector was held at 35 °C. The detection of fluoride anions occurred at 11 min, with a total runtime of 22 min.

**Table SI.7** Combustion program indicating boat positions, times and speeds for the CIC sum parameter measurements

| Position / mm | Time / s | Speed / mm/s | Position / mm | Time / s | Speed / mm/s | Position / mm | Time / s | Speed / mm/s | Position / mm | Time / s | Speed / mm/s |
| --- | --- | --- | --- | --- | --- | --- | --- | --- | --- | --- | --- |
| 65 | 30 | 10 | 100 | 60 | 0.5 | 130 | 60 | 0.5 | 150 | 60 | 0.5 |
| Position / mm | Time / s | Speed / mm/s | End time / s | Cool time / s | Home time / s | Ar time / s | O_2_ time / s |  |  |  |  |
| 259 | 100 | 20 | 10 | 60 | 120 | 10 | 600 |  |  |  |  |

**Table SI.8** Gradient and suppressor program for the IC system within the CIC sum parameter measurement

| Time / min | Eluent concentration / mM | Suppressor current / mA |
| --- | --- | --- |
| 0.0 | 1.0 | 4 |
| 0.1 | 1.0 | 4 |
| 0.2 | 2.0 | 4 |
| 1.0 | 2.0 | 4 |
| 10.0 | 5.0 | 4 |
| 10.5 | 5.0 | 4 |
| 11.0 | 80.0 | 50 |
| 14.5 | 80.0 | 50 |
| 15.0 | 1.0 | 4 |

The calculations for the fluoride levels are shown in equation SI.3:

| $Level\left( \mathrm{Fluoride} \right)=\frac{Peak area\left( Fluoride, corrected \right)-b_{CIC-calibration}}{a_{CIC-calibration}}\cdot Dilution Factors\cdot\frac{Extraction solvent volume}{Sample weight}\cdot{10}^{6}$ | **Eq.** SI.3 |
| --- | --- |

Herein, the variables and their units are as follows:

| [*Level*(Fluoride)] | ng/(g sample) |
| --- | --- |
| [*Sample weight*] | g |
| [*Peak area*(Fluoride, corrected)] | µS·min |
| [*a*_external_] | (µS·min ·L)/mg |
| [*b*_external_] | µS·min |
| [*Extraction solvent volume*] | L |
| [*Dilution Factors*] | / |

The dilution factors are composed of the dilution factor introduced by the absorption of the combusted sample in the gas absorption unit monitored by the internal standard and by the sample re-uptake in methanol subsequent to drying. The corrected peak area describes the experimentally measured peak area of the fluoride peak subtracted by the instrumental blank (average peak area of ten unloaded boat combustions from the same batch). The dilution introduced by the gas absorption unit and the peak area correction were also respected for the calibration standards.

## **SI.2 Target Method Development for PFAS and Sulfonylimides**

### **SI.2.1 HILIC-ESI-MS/MS Chromatographic Conditions**

Fig. SI. 1 shows the chromatograms of the native mix detailed in table SI.2 after separation with gradient elution vs. isocratic elution on the Raptor Polar X HILIC column and detection with the ESI-MS/MS. The gradient elution corresponds to the final method given in the main text in the experimental section. The isocratic variant was measured applying the same chromatographic and detection parameter as the method described in the method section of the main text but instead of a gradient, isocratic elution with 85 % of the organic part B and 15 % of the aqueous part A of the mobile phase was conducted and the retention times were adapted. The isocratic elution is based on the method parameter published by [3] and advertised by the column provider Restek Corporation (Bad Homburg, Germany). To achieve higher separation for all PFAS analytes treated in this work, the start conditions were adjusted to a higher organic part (95 % B instead of 85 % B at start for the original isocratic elution) because the aqueous part of the mobile phase possesses the higher elution strength in the HILIC system . The gradient from 95 % to 85 % within 6 min was added to the adjusted method to minimize peak broadening for the substances with high retention – notably TFA (PFCA C_2_). Especially in the region from 2-3 min, coelution still occurs with the adapted method. However, the sensitive and specific MS/MS detection make the method suitable for quantitative analysis in the working range validated in this work.


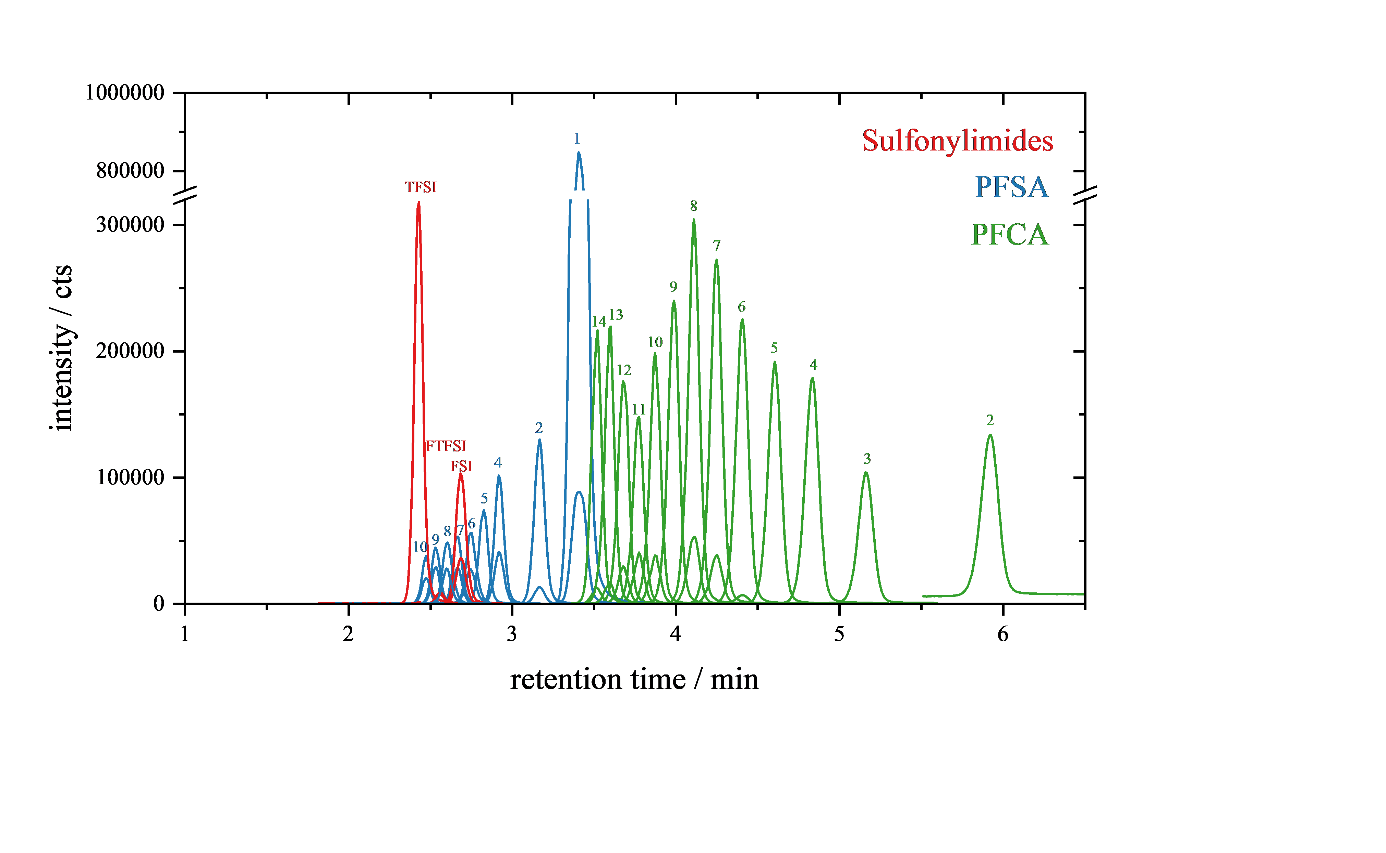

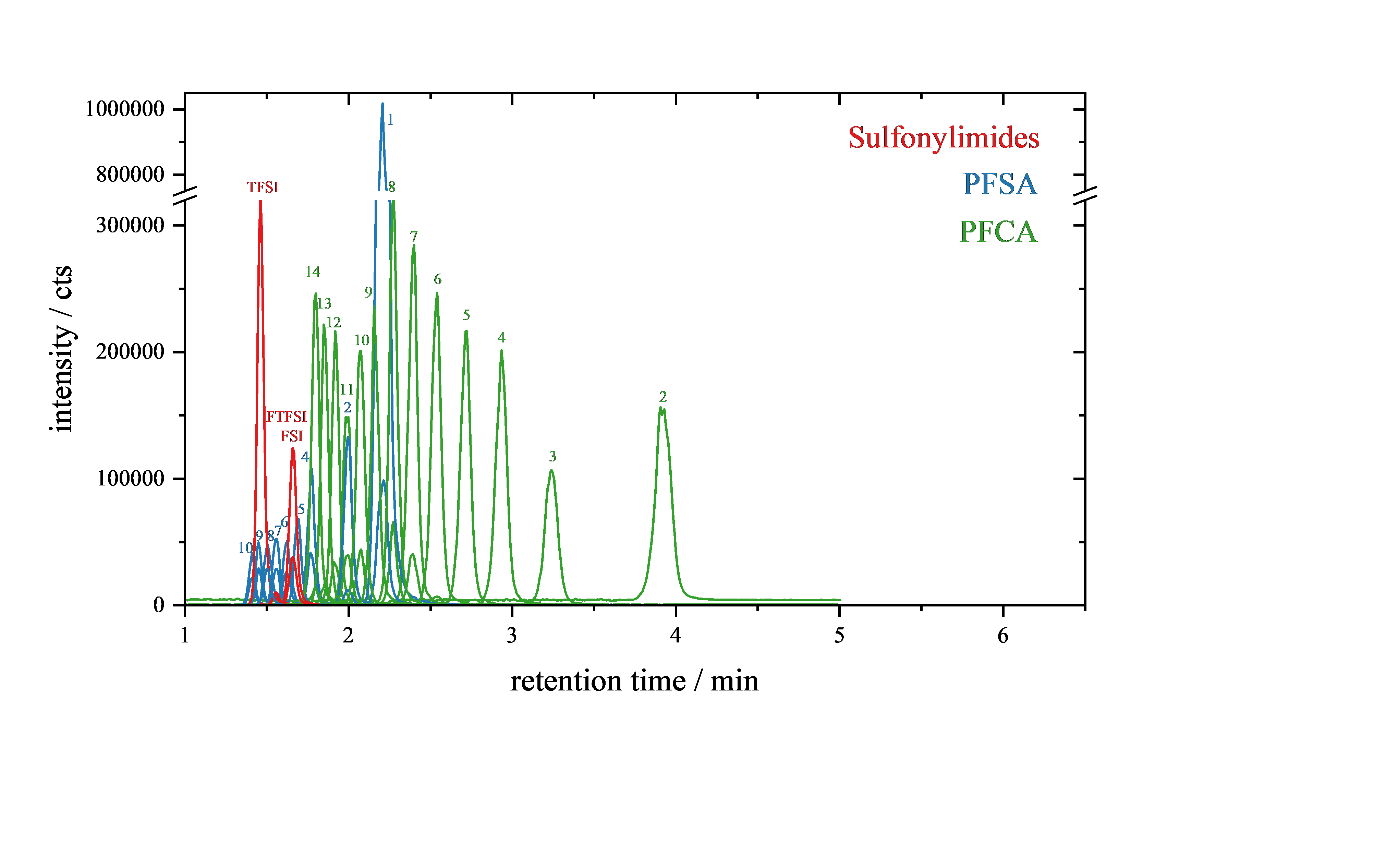


**Fig. SI. 1** Chromatograms of the native PFAS and sulfonylimide mix (see table SI.2) measured with the HILIC-ESI-MS/MS system of this work via isocratic elution (**top**) or gradient elution (**bottom**). The numbers attributed to the PFSA and PFCA peaks indicate the number of carbon atoms of the chain. The chromatographic parameter for the separation on the Raptor Polar X column are as follows: aqueous phase (A) composed by water with 0.05 % (v/v) FA and 10 mM NH_4_FA and an organic phase (B) composed by ACN/MeOH in a volumetric ratio of 60:40 with 0.05 % (v/v) FA as mobile phase at a flow rate of 0.5 mL/min with isocratic elution (85 % B, 15 % A) or gradient elution (95 % B to 85 % B in 6 min)

### **SI.2.2 Extraction Procedure**

In Fig. SI. 2, the herein used, final extraction procedure is schematically represented in case of a black mass sample, because these high matrix samples are more difficult to extract than the aqueous gas absorption solutions. The extraction starts with the leaching out of the black mass with an alkaline solution. This is supposed to solvate the analytes in deprotonated form in the aqueous solution which is probably the case for the short-chain representatives like TFA and TFMS or FSI. However, as the longer-chain PFSA and PFCA have an important organic tail, it is not sure whether they experience solvatization or stay adsorbed to the matrix. The next step represents a liquid-liquid-extraction (LLE) with MtBE to achieve a phase transfer of the analytes by saturating the aqueous solution with sodium chloride and by adding an excess of concentrated sulfuric acid. As the procedure is carried out in one-pot, the black mass sample itself is equally contacted to the MtBE which may be the key allowing to extract less polar, longer-chain analytes that may still be adsorbed to the matrix after aqueous leaching-out. The subsequent cleaning step of the organic MtBE extract ensures the minimization of salinity and unwanted particles before injection of the extract into the LC-MS/MS system.

Table SI.9 provides an overview on some of the adapted extraction method parameter (for the case of black mass samples) and their effect on the response recovery of the analytes after matrix spike on the material BM1.T compared to a reference standard in solution with identical theoretical concentration. A response recovery above 100 % indicates that the detection of the analyte is more sensitive in the extracted matrix spike than in the analyte standard. This optimization procedure allows to assure a sensitive detection of the analytes; however, matrix effects and extraction efficiency are not distinguishable through this approach and the obtained recoveries are no quantitative and exact measures. Thus, the given response recoveries do not represent a quantitative measure for the analyte recovery after extraction, unlike the recoveries represented for the method validation. The method adaptions from table SI.9 concern:

- the volume of the alkaline Na_2_CO_3_-solution for the matrix leaching out (“Na_2_CO_3_-solution”)
- the quantity of NaCl added for the salting-out within the phase transfer (“NaCl addition”)
- the quantity of concentrated H_2_SO_4_ added for the phase transfer of the analytes (“H_2_SO_4_ addition”)
-
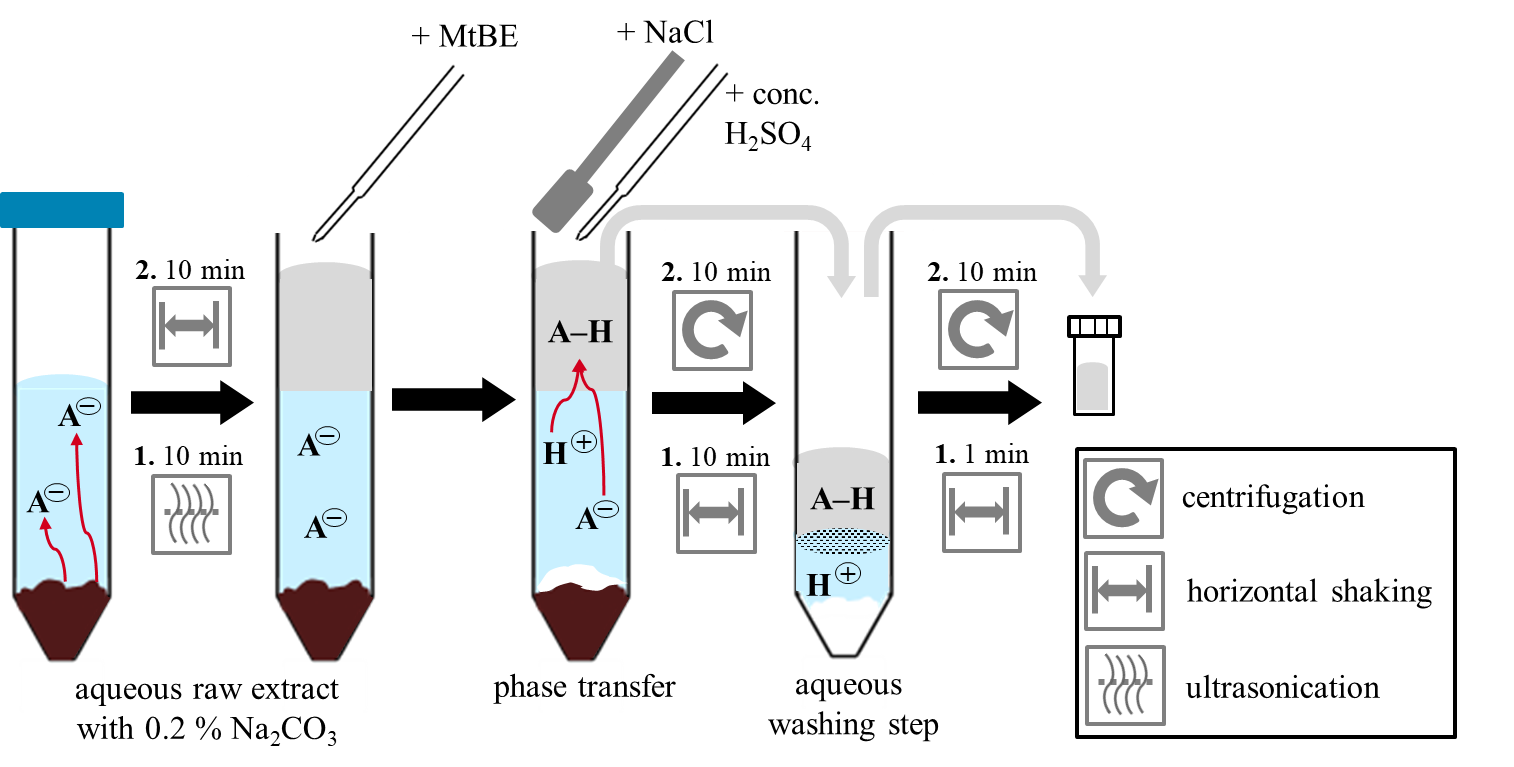
the composition of the aqueous clean-up solution for the MtBE raw extract (“clean-up solution”)

**Fig. SI. 2** Schematic representation of the sample preparation method for the black mass samples

**Table SI.9** Optimization of the extraction method regarding response recovery of the analytes spiked on matrix compared to a reference standard with identical theoretical concentration

| Method adaption | 1 | 2 | 3 (=final method) |
| --- | --- | --- | --- |
| Na_2_CO_3_-solution | 8 mL | 6 mL | 6 mL |
| NaCl addition | 1.5 | 2 | 2 |
| H_2_SO_4_ addition | 120 | 180 | 200 |
| clean-up solution | 2 % H2SO4(aq) | 5 % H2SO4(aq) + 1g NaCl | 18 % H2SO4(aq) + 1 g NaCl |
| Analyte | Response recovery / % | | |
| TFSI | 82 | 97 | 144 |
| FTFSI | 50 | 95 | 137 |
| FSI | 104 | 114 | 131 |
| PFDS | 106 | 114 | 136 |
| PFNS | 110 | 137 | 145 |
| PFOS | 101 | 123 | 146 |
| PFHpS | 102 | 127 | 143 |
| PFHxS | 77 | 97 | 145 |
| PFPeS | 79 | 105 | 144 |
| PFBS | 69 | 95 | 139 |
| PFEtS | 0 | 73 | 99 |
| TFMS | / * | / * | 16 |
| PFTDA | 124 | 140 | 146 |
| PFTrDA | 110 | 137 | 153 |
| PFDoDA | 102 | 138 | 162 |
| PFUnDA | 103 | 140 | 191 |
| PFDA | 103 | 137 | 181 |
| PFNA | 84 | 121 | 160 |
| PFOA | 77 | 114 | 172 |
| PFHpA | 67 | 111 | 166 |
| PFHxA | 80 | 115 | 172 |
| PFPeA | 93 | 107 | 138 |
| PFBA | 120 | 117 | 116 |
| PFPrA | / * | / * | 120 |
| TFA | 304 | 252 | 158 |

* Not yet available as standards during method testing.

By optimizing the given parameter of the extraction (compare table SI.9), the amount of sulfuric acid – for phase transfer and present in the aqueous solution for the cleaning step – was found to be critical for high response recoveries (> 100 % for all analytes except TFMS with 16 %, see SI table SI.9). It can be imagined that the addition of sulfuric acid protonates the analytes and increases their tendency to migrate into the organic MtBE phase. However, given the theoretical p*K*_a_ values of the analytes [4-6], for the PFSA and the sulfonylimides, the protonation by sulfuric acid is doubtable. Especially TFMS and TFSI are often referred to as super acids [7]. This may explain TFMS showing weak response recoveries with the final extraction method, indicating that it either suffers from matrix suppression or a non-efficient extraction. Nevertheless, for the longer-chain representatives and the sulfonylimides, high response recoveries are achieved anyways which may be due to the salting-out procedure and to the high affinity of these analytes to partition into organic phases [8]. These observations leads to the conclusion that the extraction may rely on a combination of analyte p*H* conditions and partition preferences of the analytes.

Globally, the herein shown extraction method represents a quick (approximatively < 3 hours per batch of 15-20 samples) and easy approach to prepare solid or liquid samples for subsequent analysis with LC-MS/MS methods compatible with organic solvents. The sensitive detection of analytes can be expected, except for TFMS, making the method suitable for quantitative analysis.

## **SI.3 LC-MS/MS Instrumentation Incorporating the Hexafluorophosphate Anion**

### **SI.3.1 Method Development for HILIC-ESI-MS/MS with the Raptor Polar X Column**

Fig. SI. 3 shows the chromatogram of FSI- and hexafluorophosphate (PF6)-standards after separation on the Raptor Polar X column with an adapted chromatographic method. With the HILIC-ESI-MS/MS method used for the PFAS and sulfonylimides, FSI and PF6 co-eluted at 2.7 min leading to suppression of the PF6 signal in black mass matrix samples. For the quantification of PF6, separation of both compounds present in the black masses was therefore necessary. Different chromatographic parameter (buffer concentration, flow rate, MeOH/ACN ratio in the organic part of the mobile phase) were tested. Amongst those, the MeOH percentage was found to be crucial for increasing the retention of FSI and PF6 leading to a modified selectivity and separation of both. Based on these findings, the separation method was adjusted to the conditions described in the following section.





**Fig. SI. 3** Chromatogramm of a standard containing FSI (40 ng/(g solvent)) and PF6 (245 ng/(g solvent)) in MeOH with the HILIC-ESI-MS/MS method adapted for their separation. The chromatographic parameter for the separation on the Raptor Polar X column are as follows: aqueous phase (A) composed by water with 0.05 % (v/v) FA and 10 mM NH_4_FA and an organic phase (B) composed by ACN/MeOH in a volumetric ratio of 20:80 with 0.05 % (v/v) FA as mobile phase at a flow rate of 0.4 mL/min with isocratic elution (85 % B, 15 % A)

### **SI.3.2 Instrumentation of the Final Method via HILIC-ESI-MS/MS with the Raptor Polar X Column**

LC-MS/MS measurements were performed using an Agilent 1290 Infinity II LC system (Agilent Technologies Deutschland GmbH, Waldbronn, Germany) coupled to an Agilent 6495C triple quadrupole MS (Agilent Technologies Deutschland GmbH, Waldbronn, Germany) equipped with an electrospray ionization (ESI) spray chamber (Agilent Jet Stream). The system was controlled by the Mass Hunter Workstation (version 10.1, Agilent Technologies Deutschland GmbH).

The injection of the principally in MeOH dissolved samples was set to a volume of 0.5 µL and carried out with by an automated sampler. Chromatographic separation was achieved with a Raptor Polar X HILIC column (2.1 mm x 50 mm; 2.7 µm particles; Restek Corporation, Bad Homburg Germany) equipped with a Raptor Polar X guard column (2.1 mm x 5 mm; Restek Corporation, Bad Homburg Germany). The column temperature was set to 40 °C and the flow rate to 0.4 mL/min. A mixture of an aqueous phase (A) composed by water with 0.05 % (v/v) FA and 10 mM NH_4_FA and an organic phase (B) composed by ACN/MeOH in a volumetric ratio of 20:80 with 0.05 % (v/v) FA was used as mobile phase in isocratic mode. Nitrogen was used as dry gas at a flow rate of 11 L/min and a temperature of 250 °C. The nebulizer pressure was set to 25 psi and the capillary voltage was set to 3 kV in negative ion mode. The detection was performed in a targeted dynamic multiple reaction monitoring (dMRM) mode with a 1 min retention time window for each analyte and a cycle time of 500 ms. The fragmentor voltage was fixed at 166 V and the collision energy was set to 43 eV. The detection of hexafluorophosphate was possible at a pseudo-mass transition with m/z 145 to m/z 145 at a retention time of 5.0 min. The calibration for PF6 was performed via an ten-point external calibration (*R*^2^=0.99565) with diluted reference standards in MeOH produced from a 1 M LiPF_6_ electrolyte solution at the levels: 0, 1, 8, 35, 126, 245, 363, 474, 583, 682 ng/(g solvent). The calibration curve data are given in table SI.10. All other target analytes measured along with hexafluorophosphate with the adapted chromatographic conditions were detected following the MS/MS parameter described in the original method and detailed in table SI.3.

### **SI.3.3 Quantifications and Updated Fluorine Mass Balance Including Hexafluorophosphate**

Table SI.10 shows the calibration curve data for the external calibration for the hexafluorophosphate (PF6) anion.

**Table SI.10** External calibration data for all analytes with the HILIC-ESI-MS/MS procedure and after preparation of the calibration standards according to the sample preparation

| Native analyte | Number of calibration standards | Calibration curve slope *a*_external_ / (cts·s·g)/ng | Calibration curve *y*-intersection *b*_external_ / cts·s | Calibration curve *R*^2^ | Level lowest calibration point / ng/(g solvent) in MeOH | Level highest calibration point / ng/(g solvent) in MeOH |
| --- | --- | --- | --- | --- | --- | --- |
| PF6 | 10 | 167 | 740 | 0.99565 | 1 | 682 |

Table SI.11 contains the levels of PF6 extracted from the examined black mass samples and presents their contribution to the fluorine sum parameter.

**Table SI.11** Mass balance of the target analytes and the extractable fluorine sum parameter for the black mass materials

| **Material** | **Extractable fluorine sum parameter *^1^ / ng/(g sample)** | | **Level PF6 *^2^ /**  **ng/(g sample)** | **Fluorine equivalents *^3^/  ng/(g sample)** | | | | **Non-explainable extractable fluorine / %** |
| --- | --- | --- | --- | --- | --- | --- | --- | --- |
|  |  |  |  | **PF6** | **FSI *^4^** | **PFBS** | **TFA** |  |
| BM1 | 4600 ± 50 | 96 | | 76 | 68 ± 1 | 4.0 ± 0.6 | - | 96.8 |
| BM1.T | 177 ± 6 | - | | - | - | - | - | n.a. *^5^ |
| BM2 | 8500 ± 80 | 295 | | 232 | 157 ± 6 | 3.2 ± 0.6 | 0.308 ± 0.007 | 95.4 |

*^1^ The fluorine sum parameter is calculated based on one independent sample extraction with three-fold injection. The values are corrected against the instrument blank but not against the method blank which is at 297 ± 18 ng/(g sample) extractable fluorine.

*^2^ The PF6 level was determined in an aliquot of the respective sample used for the CIC sum parameter measurement to minimize bias and is therefore indicated without uncertainty.
*^3^ All quantified analyte levels are based on independently prepared triplicates and valuable for the protonated analyte form unless indicated differently. The fluorine equivalents respect the loss of analyte due to the drying and re-uptake in MeOH of the extracts for the CIC sum parameter measurements.
*^4^ Quantified in the black mass samples via an external calibration due to high levels. The quantifications are valuable for the deprotonated analyte form.
*^5^ Not applicable, because the fluorine sum parameter is below the method blank indicating that the sample can be considered as uncontaminated with extractable fluorine.

### **SI.3.4 Hexafluorophosphate Related Fluorinated Degradation Products**

Fig. SI.3 shows the chromatograms of an extract of the sample BM1 diluted with MeOH by the factor 4 and of the extract used for the CIC sum parameter measurement after drying and re-uptake in MeOH. Both extracts were measured with the HILIC-ESI-MS/MS method adapted for PF6 (described in SI.3.2) extended to a run time of 13.5 min and with incorporation of the mass transitions with the highest intensities detected in low resolution scans.

At retention times >7 min, masses with transitions to m/z 79 (PO_3_^-^) and m/z 63 (PO_2_^-^) or transitions involving the m/z 99 (PO_3_HF^-^) occurred which can be related to (fluorinated) phosphate species or (fluorinated) phosphoric acid esters. Table SI.12 shows the mass transitions observed at identical retention times in both extracts. Based on the mass transitions and on comparison with literature work on the degradation of PF6 [9], species at the origin of the measured signals are postulated.

Fig. SI. 4 also shows, that the PF6 peak decreases with the drying and re-uptake procedure which is why quantifications for the fluorine mass balance needed to be performed on an extract treated in the same manner as the one used for the CIC sum parameter measurements. This underlines the unstable chemical nature of PF6. Thus, it cannot be excluded that some of the postulated degradation products do not originate from the black mass matrix itself but are formed only during the extraction.








**Fig. SI. 4** Chromatograms of black mass (BM1) extracts measured with the HILIC-ESI-MS/MS method adapted to PF6 and including highly intensive mass transitions discovered in low resolutions scans. **Left**: Extract of the sample BM1 diluted with MeOH by the factor 4; **Right**: Extract used for the CIC sum parameter measurement after drying and re-uptake in MeOH with no significant dilution. The chromatographic parameter for the separation on the Raptor Polar X column are as follows: aqueous phase (A) composed by water with 0.05 % (v/v) FA and 10 mM NH_4_FA and an organic phase (B) composed by ACN/MeOH in a volumetric ratio of 20:80 with 0.05 % (v/v) FA as mobile phase at a flow rate of 0.4 mL/min with isocratic elution (85 % B, 15 % A) with a runtime of 13.5 min. The fragmentor voltage was set to 166 V and the collision energy for the transitions with retention times > 7 min was fixed at 43 eV

**Table SI.12** Observed mass transitions in black mass extracts and possible fluorinated phosphate or phosphoric acid ester species at their origin (compare Fig. SI.3)

|  | Retention time / min | m/z → m/z | m/z → m/z | m/z → m/z | m/z → m/z | Postulated substance in comparison with Kraft *et al.* [9] |
| --- | --- | --- | --- | --- | --- | --- |
|  |  | (Collision energy 43 eV for all mass transitions) | | | |  |
| A’ | 7.4 | 101 (PO_2_F_2_^-^) → 63 (PO_2_^-^) | 101 (PO_2_F_2_^-^) → 101 (PO_2_F_2_^-^) |  |  | Difluoro phosphate |
| B’ | 8.4 | 99 (PO_3_HF^-^) → 79 (PO_3_^-^) | 99 (PO_3_HF^-^) → 63 (PO_2_^-^) | 157 →  79 (PO_3_^-^) | 157 →  82 (PO_2_F^-^) | Unknown *^1^ |
| C’ | 9.0 | 99 (PO_3_HF^-^) → 79 (PO_3_^-^) | 99 (PO_3_HF^-^) → 63 (PO_2_^-^) |  |  | Unknown *^2^ |
| D’ | 9.9 | 141 (M^-^) →  79 (PO_3_^-^) | 141 (M^-^) →  63 (PO_2_^-^) | 99 (PO_3_HF^-^) → 79 (PO_3_^-^) | 99 (PO_3_HF^-^) → 63 (PO_2_^-^) | Unknown *^3^ |
| E’ | 11.2 | 127 (M^-^) →  79 (PO_3_^-^) | 127 (M^-^) →  99 (PO_3_HF^-^) | 99 (PO_3_HF^-^) → 79 (PO_3_^-^) | 99 (PO_3_HF^-^) → 63 (PO_2_^-^) | Ethyl fluorophosphate |
| F’ | 12.5 | 113 (M^-^) →  79 (PO_3_^-^) | 113 (M^-^) →  98 (PO_3_F·^-^) |  |  | Methyl fluorophosphate |

*^1^ Due to the occurrence of the m/z 157, methoxyethyl fluorophosphate could be at the origin of the signal, but the related signals are very low in intensity.

*^2^ Can not be assigned to a specific molecule due to the lack of a particular mass transition distinguishing the signal from other fluorinated phosphates or phosphoric acid ester. Thus, the signal could belong to fluorophosphate (PO_3_HF^-^), but this remains an unconfirmed hypothesis.

*^3^ Due to the occurrence of the m/z 141, the M^-^ could be propyl fluorophosphate, which was not described by Kraft *et al.* or the signal represents another unknown species.

1. Roesch P, Vogel C, Huthwelker T, Wittwer P, Simon F-G. Investigation of per- and polyfluoroalkyl substances (PFAS) in soils and sewage sludges by fluorine K-edge XANES spectroscopy and combustion ion chromatography. Environmental Science and Pollution Research. 2022;29:26889–99.

2. Vogel C, Roesch P, Wittwer P, Sommerfeld T, Riedel M, Leube P, et al. Per- and polyfluoroalkyl substances (PFAS) in Ski waxes and snow from cross-country skiing in Germany - Comparative study of sum parameter and target analysis. Journal of Hazardous Materials Advances. 2024;16:100484.

3. Liang S-H, Chakraborty M, Steimling JA. Incorporating ultrashort-chain compounds into the comprehensive analysis of per- and polyfluorinated substances in potable and non-potable waters by LC-MS/MS. Journal of Chromatography Open. 2024;6:100188.

4. Rayne S, Forest K. Estimated pKa values for the environmentally relevant C1 through C8 perfluorinated sulfonic acid isomers. Journal of Environmental Science and Health, Part A. 2016;51(12):1018–23.

5. Rayne S, Forest K. Theoretical studies on the pK~a~ values of perfluoroalkyl carboxylic acids. Nature Precedings. 2010.

6. Chipanina NN, Sterkhova IV, Aksamentova TN, Sherstyannikova LV, Kukhareva VA, Shainyan BA. Structure of bis(trifluoromethanesulfonyl)imide in inert and protophilic media. Russian Journal of General Chemistry. 2008;78(12):2363–73.

7. Gal J-F, Iacobucci C, Monfardini I, Massi L, Duñach E, Olivero S. Metal triflates and triflimides as Lewis “superacids”: preparation, synthetic application and affinity tests by mass spectrometry. Journal of Physical Organic Chemistry. 2013;26(2):87–97.

8. Rayne S, Forest K. An assessment of organic solvent based equilibrium partitioning methods for predicting the bioconcentration behavior of perfluorinated sulfonic acids, carboxylic acids, and sulfonamides. Nature Precedings. 2009.

9. Kraft V, Grützke M, Weber W, Winter M, Nowak S. Ion chromatography electrospray ionization mass spectrometry method development and investigation of lithium hexafluorophosphate-based organic electrolytes and their thermal decomposition products. Journal of Chromatography A. 2014;1354:92–100.
